# Nucleophilic Substitution at Tetracoordinate Phosphorus. Stereochemical Course and Mechanisms of Nucleophilic Displacement Reactions at Phosphorus in Diastereomeric *cis*- and *trans*-2-Halogeno-4-methyl-1,3,2-dioxaphosphorinan-2-thiones: Experimental and DFT Studies

**DOI:** 10.3390/molecules26123655

**Published:** 2021-06-15

**Authors:** Marian Mikołajczyk, Barbara Ziemnicka, Jan Krzywański, Marek Cypryk, Bartłomiej Gostyński

**Affiliations:** 1 Department of Organic Chemistry, Centre of Molecular and Macromolecular Studies, Polish Academy of Sciences, Sienkiewicza 112, 90-363 Łódź, Poland; bziemnicka@cbmm.lodz.pl (B.Z.); jan.krzywanski658@gmail.com (J.K.); 2 Department of Structural Chemistry, Centre of Molecular and Macromolecular Studies, Polish Academy of Sciences, Sienkiewicza 112, 90-363 Łódź, Poland; bgostyns@cbmm.lodz.pl

**Keywords:** 2-halogeno-4-methyl-1,3,2-dioxaphosphorinan-2-thiones, alkaline hydrolysis, mechanism, stereochemistry, DFT calculations

## Abstract

Geometrical *cis*- and *trans*- isomers of 2-chloro-, 2-bromo- and 2-fluoro-4-methyl-1,3,2-dioxaphosphorinan-2-thiones were obtained in a diastereoselective way by (a) sulfurization of corresponding cyclic P^III^-halogenides, (b) reaction of cyclic phosphorothioic acids with phosphorus pentachloride and (c) halogen–halogen exchange at P^IV^-halogenide. Their conformation and configuration at the C_4_-ring carbon and phosphorus stereocentres were studied by NMR (^1^H, ^31^P) methods, X-ray analysis and density functional (DFT) calculations. The stereochemistry of displacement reactions (alkaline hydrolysis, methanolysis, aminolysis) at phosphorus and its mechanism were shown to depend on the nature of halogen. Cyclic *cis*- and *trans*-isomers of chlorides and bromides react with nucleophiles (HO^−^, CH_3_O^−^, Me_2_NH) with inversion of configuration at phosphorus. DFT calculations provided evidence that alkaline hydrolysis of cyclic thiophosphoryl chlorides proceeds according to the S_N_2-P mechanism with a single transition state according to the potential energy surface (PES) observed. The alkaline hydrolysis reaction of *cis*- and *trans*-fluorides afforded the same mixture of the corresponding cyclic thiophosphoric acids with the thermodynamically more stable major product. Similar DFT calculations revealed that substitution at phosphorus in fluorides proceeds stepwise according to the A–E mechanism with formation of a pentacoordinate intermediate since a PES with two transition states was observed.

## 1. Introduction

**Figure 1:**
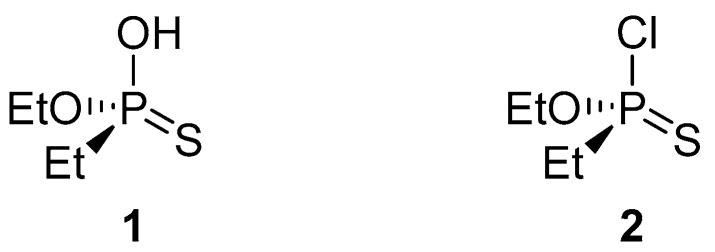
Structures of optically active acyclic thiophosphonic acid (**1**) and the corresponding thiophosphonyl chloride (**2**) used in studies of nucleophilic substitution at phosphorus.

The stereochemistry and mechanisms of nucleophilic substitution reactions at the phosphorus atom have been the major points of interest of many research groups since the middle of the last century [1]. The stereochemical course of nucleophilic displacement reactions at phosphorus has also been a subject of our early systematic studies, which have employed, in the first stage, optically active acyclic thiophosphonic acids **1** and their derivatives, as for example the enantiomeric thiophosphonyl chlorides **2** (Figure 1).

These early investigations resulted, among others, in elaboration of a Walden cycle for thiophosphonic acids **1** (interconversion of the enantiomers of **1**) [2,3] and in providing unequivocal proof for the inversion of configuration at phosphorus occurring in the chloride exchange reaction between radioactive chloride anion (^36^Cl^−^) and optically active thiophosphonyl chloride **2** [4]. It has also been demonstrated that alkaline hydrolysis of optically active chlorides **2** proceeds with inversion at the P atom [5].

**Scheme 1:**
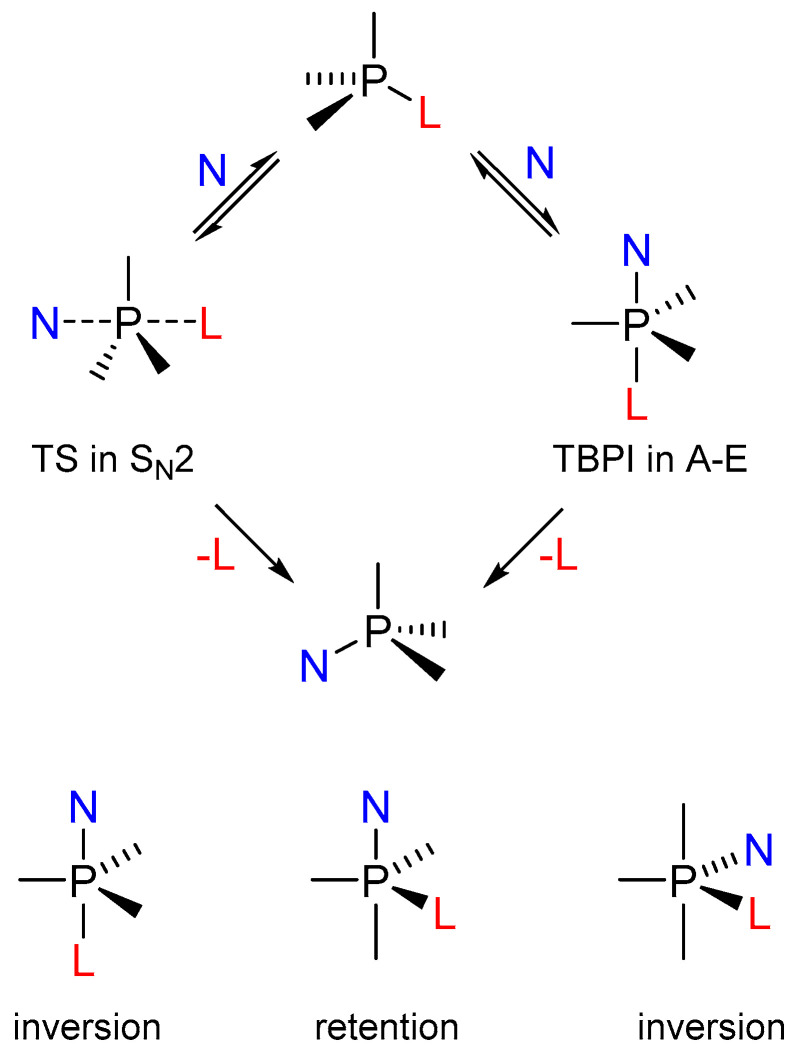
Mechanisms of nucleophilic substitution reactions at phosphorus and structures of possible pentacoordinate phosphorus intermediates (TBPI) formed by the A–E mechanism.

The second most important question about the mechanisms of displacement reactions at phosphorus was, and it is still, whether these reactions occur synchronously according to the S_N_2-P mechanism via a single transition state (TS) or stepwise by the addition–elimination mechanism (A–E) involving formation of an unstable, pentacoordinate phosphorus intermediate (trigonal bipyramidal intermediate, TBPI). It is formed in the first reaction step by addition of the nucleophilic reagent (N) to the electrophilic phosphorus substrate and decomposes in the second step by the departure of the leaving group (L) to the reaction product (see Scheme 1).

In analogy with the S_N_2 substitution at carbon, the S_N_2-P reactions also occur with inversion of configuration. However, the relationship between the stereochemistry and the A–E mechanism is more complicated. It is now generally accepted that the stereochemical outcome of such stepwise reactions depends mainly on the disposal of the attacking N and leaving L groups in a short-living intermediate TBPI. Thus, the diapical or diequatorial disposal of N and L should lead to inversion of configuration at phosphorus while the stereochemical outcome of the apical-equatorial substitution is predicted to be retention. Moreover, these three different TBPIs may be formed as primary intermediates or as a result of their pseudorotation. In this connection, it is necessary to emphasize a great importance of the Westheimer’s concept [6] and his rules (apical entry of N, apical departure of L, microscopic reversibility and pseudorotation of TBPI) for understanding many aspects of nucleophilic substitution at phosphorus as well as at other heteroatoms such as sulfur [7]. The results of his careful studies on acid-catalyzed bidirectional hydrolysis of methyl ethylene phosphate **3** (2-methoxy-1,3,2-dioxaphospholan-2-one) and the concurrent rapid ^16^O/^18^O oxygen exchange in the phosphoryl group of **3** provided arguments for the A–E mechanism involving transient formation of a trigonal bipyramidal intermediate (TBPI-a) with the five-membered ring occupying the energetically most favorable apical-equatorial position and its Berry pseudorotation (BPR).

**Scheme 2:**
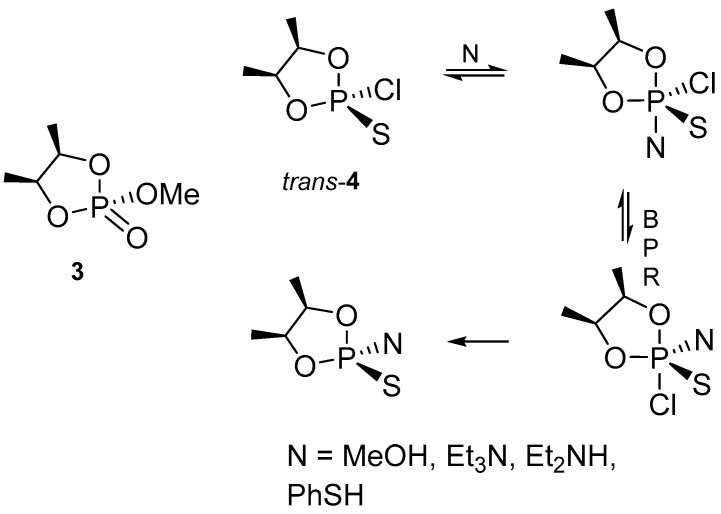
Structure of methyl ethylene phosphate **3** and stereochemical course of displacement reactions at phosphorus in *trans*-2-chloro-4,5-dimethyl-1,3,2-dioxaphospholan-2-thione **4**.

Although methyl ethylene phosphate **3** is an achiral molecule, an inspection of the pathway for the external hydrolysis of **3** and the oxygen ^16^O/^18^O exchange proposed by Westheimer strongly suggested that in cyclic five-membered phosphorus compounds the nucleophilic displacement reactions should occur with retention of configuration at P. This was found to be the case [8]. Using the geometrical *cis* and *trans* isomers of 2-chloro-4,5-dimethyl-1,3,2-dioxaphospholan-2-thiones **4** as model compounds, we have demonstrated that the replacement of chloride anion in *trans*-**4** by nucleophilic reagents N takes place with retention of configuration at phosphorus. This stereochemical outcome of the reaction was best rationalized in terms of the A–E mechanism shown in Scheme 2 and provided experimental, stereochemical evidence in support of the Westheimer’s concept.

**Figure 2:**
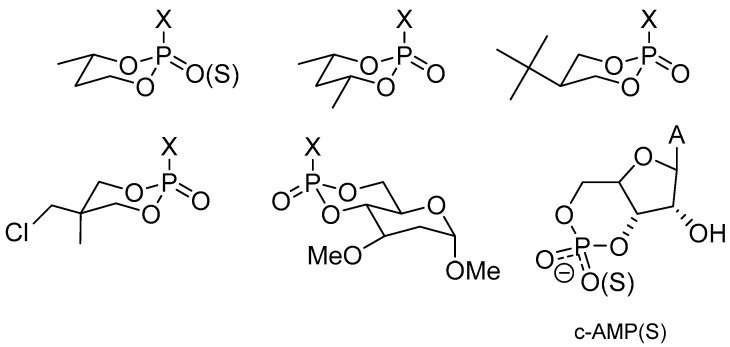
Structures of 1,3,2-dioxaphosphorinanes most often used in studies of displacement reactions at phosphorus.

In the case of cyclic five-membered phosphorus compounds (1,3,2-dioxaphospholanes), the ring occupies apical and equatorial position in the intermediate adduct (TBPI) since its diequatorial arrangement would cause a large steric strain [6]. This led to the question of the effect of a six-membered ring in 1,3,2-dioxaphosphorinanes on the stereochemical course and the mechanism of substitution at phosphorus. In this case, the diequatorial or apical-equatorial positions of the ring in the TBPI are equally probable and are not connected with the essential strain energy changes [9].The stereochemistry of the displacement reactions at phosphorus was intensively investigated using the geometrical *cis*- and *trans*-isomers of the structurally diverse 1,3,2-dioxaphosphorinanes shown in Figure 2.In this place, it is interesting to note that 1,3,2-dioxaphosphorinanes may be considered as oversimplified analogues of cyclic nucleotides-adenosine 3′,5′-phosphate (c-AMP) and thiophosphate (c-AMPS). The elucidation of the mechanism of substitution at phosphorus could shed light on enzymatic hydrolytic processes of cyclic nucleotides.

The results of these very extensive studies on the stereochemistry of nucleophilic substitution reactions at phosphorus in 1,3,2-dioxaphosphorinanes, including also the synthesis of geometrical isomers and determination of their conformation, were summarized in a comprehensive review paper entitled “Stereochemical Aspects of Phosphorus-Containing Cyclohexanes” published in 1979 [10].

In general, the displacement reactions in 1,3,2-dioxaphosphorinanes investigated were found to occur with both inversion and retention at phosphorus. The stereochemical outcome depended on many factors like the nature of the entering and leaving group, solvent, added salts and reaction conditions. However, in spite of the fact that rich experimental material had been accumulated, it was not possible at that time to draw firm conclusions on the mechanism of the displacement reactions investigated. The results were explained by assuming operation of either the S_N_2 or A–E mechanisms. The best recapitulation of the results obtained in this early period may be found in one of the many papers devoted to the problem and is as follows: “The interpretation of our results is not a simple matter and should be accepted only as a starting point for further investigations” [11].

**Figure 3:**
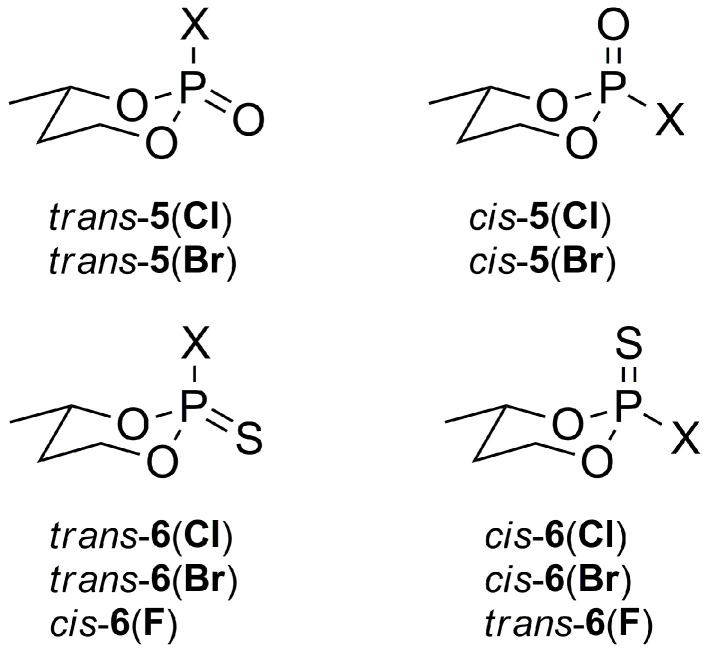
Structures of diastereomeric 2-halogeno-4-methyl-1,3,2-dioxaphosphorinan-2-ones (**5**) and 2-thiones (**6**) prepared and investigated by our group.

In our studies on the nucleophilic substitution reactions at phosphorus, the *cis*- and *trans*-isomers of 2-chloro- and 2-bromo-4-methyl-1,3,2-dioxaphosphorinan-2-ones (**5**) were used as model compounds (Figure 3).

It was found that methanolysis and aminolysis occur with a full or predominant inversion of configuration [12]. The discussion of a possible mechanism of substitution at P was not conclusive and typical for all the papers at that time. Then, we prepared a new series of diastereomeric cyclic thiophosphoryl halogenides: *trans*-2-chloro-, *trans*-2-bromo- and *cis*-2-fluoro-4-methyl-1,3,2-dioxaphosphorinan-2-thiones **6**. In contrast to cyclic *trans*-chloride **6**(**Cl**) and *trans*-bromide **6**(**Br**), which reacted with nucleophilic reagents with inversion of configuration at phosphorus, *cis*-fluoride **6**(**F**) was found to give, upon treatment with nucleophiles, a mixture of the corresponding isomeric products, the major one being formed with retention at phosphorus. These preliminary results reported in two short communications [13,14] did not allow us to unequivocally distinguish between a concerted S_N_2-P substitution and an addition–elimination (A–E) mechanism. In general, it is difficult or impossible to make this distinction based only on stereochemical results. For instance, the observation of inversion of configuration at phosphorus may be explained not only by the S_N_2-P mechanism but also by the A–E mechanism provided that the primarily formed trigonal bipyramidal phosphorane intermediate (TBPI) with nucleophilic reagent (N) and leaving groups (L) occupying apical positions undergoes decomposition to substitution product faster than pseudorotation.

Quite recently, we were able to solve these fundamental mechanistic questions in parallel studies on nucleophilic substitution at tetracoordinate sulfur [15]. We found that the results of kinetic studies of the chloride–chloride exchange reaction in arenesulfonyl chlorides in combination with the DFT computations of this identity reaction gave convincing evidence for operation of the S_N_2-S mechanism with a single transition state. Similar DFT calculations for the fluoride exchange reaction in sulfonyl fluorides revealed that it proceeds stepwise according to the A–E mechanism with the formation of a pentacoordinate sulfur intermediate since a triple-well energy profile with two transition states was observed. The results mentioned above encouraged us to combine our stereochemical studies with theoretical calculations to definitely solve the question of the mechanism of substitution reactions at phosphorus (S_N_2-P or A–E) in isomeric 2-halogeno-4-methyl-1,3,2-dioxaphosphorinan-2-thiones **6**. In the present paper we report the complete results of our studies on this class of compounds, including their stereospecific synthesis, stereochemical course of displacement reactions at phosphorus in **6** and the DFT calculations aimed at determination of the mechanisms of the substitution reactions.

## 2. Results and Discussion

### 2.1. Synthesis, Conformation and Configuration of Diastereomeric Trans- and cis-2-Halogeno-4-methyl-1,3,2-dioxaphosphorinan-2-thiones **6**

Our initial investigations were focused on the efficient preparation and assignment of stereochemistry (ring conformation, configuration of the C_4_ and phosphorus stereocentres) to 2-chloro-, 2-bromo- and 2-fluoro-4-methyl-1,3,2-dioxaphosphorinan-2-thiones **6**, which were intended to use as model compounds in subsequent studies of the stereochemistry of nucleophilic substitution at phosphorus.

**Scheme 3:**
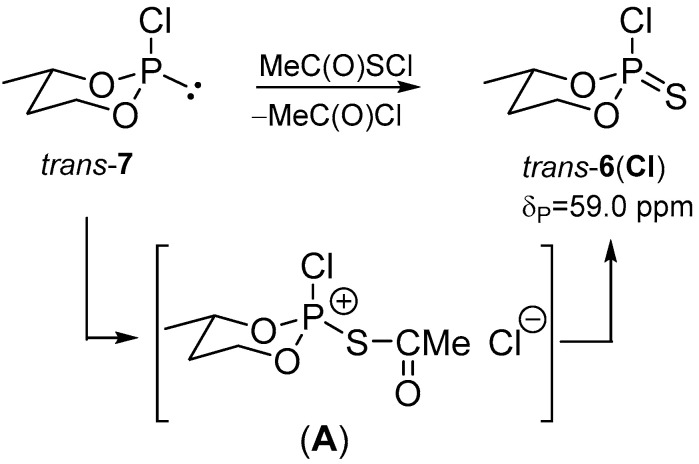
Stereospecific synthesis of *trans*-2-chloro-4-methyl-1,3,2-dioxaphosphorinan-2-thione **6**(**Cl**).

At first, *trans*-2-chloro-4-methyl-1,3,2-dioxaphosphorinan-2-thione **6**(**Cl**) was prepared by treatment of *trans*-2-chloro-4-methyl-1,3,2-dioxaphosphorinan **7** [16,17,18] with acetylsulfenyl chloride. The reaction was carried out in ether at 0 °C and afforded *trans*-**6**(**Cl**) as a pure diastereomer (^31^P-NMR assay) in 91% yield (Scheme 3).

This reaction, as other reactions of P^III^-compounds with sulfenyl chlorides [19], gave the desired product **6**(**Cl**) in a stereospecific way with retention of configuration at phosphorus via the intermediate quasi-phosphonium chloride (**A**). Hence, the isomer of **6**(**Cl**) obtained has the same *trans*-relation between the C_4_-methyl group and the chlorine atom as in the starting phosphorochloridite (**7**).

To gain more detailed insight into the ring stereochemistry and conformation the 270 MHz ^1^H-NMR spectrum of *trans*-**6**(**Cl**) was recorded. It showed that all the resonance signals of the ring protons were well separated from each other, thus allowing the first order analysis of the spectrum without use of spin decoupling. In Table 1, all of the chemical shifts and coupling constants, derived from the spectrum, are summarized.

**Figure 4:**
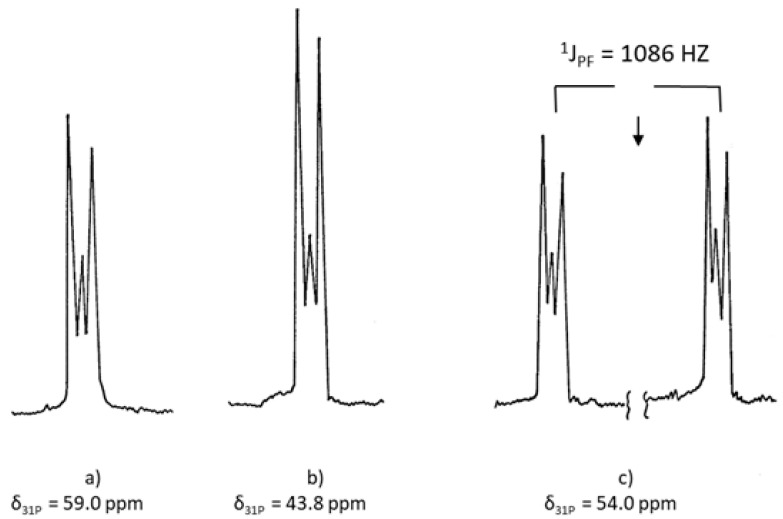
^31^P-NMR spectra of diastereomeric 2-halogeno-4-methyl-1,3,2-dioxaphosphorinan- 2-thiones **6** obtained with a Jeol-C60H-NMR spectrometer; (**a**) *trans*-**6**(**Cl**) (**b**) *trans*-**6**(**Br**) and (**c**) *cis*-**6**(**F**).

These values and particularly very distinct differences between the long-range ^31^P-^1^H couplings observed for axial and equatorial protons indicated that the dioxaphosphorinan ring in *trans*-**6**(**Cl**) adopts the chair conformation with the equatorial C_4_-methyl group and thiophosphoryl sulfur. Thus, the methyl protons appeared in the spectrum as a double doublet with coupling constants with the axial methine proton, ^3^*J*_CH3-CH_ = 6.2 Hz, and with phosphorus, ^4^*J*_CH3-P_ = 2.6 Hz. These values are diagnostic for equatorial position of the methyl group in the dioxaphosphorinan ring. The ^31^P-NMR spectrum of *trans*-**6**(**Cl**) showed the resonance signal shaped like a double multiplet at δ_P_ = 59.0 ppm (see Figure 4, which is characteristic and typical for the 1,3,2-dioxaphosphorinan ring with the methyl group and thiophosphoryl sulfur in equatorial positions.) Interestingly, almost the same shape of the ^31^P-spectrum was observed for the phosphoryl analogues of our compounds [12,20].

The second, convenient approach to the same isomer *trans*-**6**(**Cl**) consists in chlorination of an equimolar mixture of *cis*- and *trans*-2-hydrogen-4-methyl-1,3,2-dioxaphosphorinan-2-thiones **8 [21]** by means of sulfuryl chloride. The chlorination reaction was performed in benzene at 0 °C and afforded, rather unexpectedly, a diastereomerically pure *trans*-**6**(**Cl**) in 80% yield. However, when the reaction was carried out at −40 °C, a mixture of *trans*-**6**(**Cl**) (85%) and *cis*-**6**(**Cl**) (15%) was formed as revealed by ^31^P-NMR spectra.

**Scheme 4:**
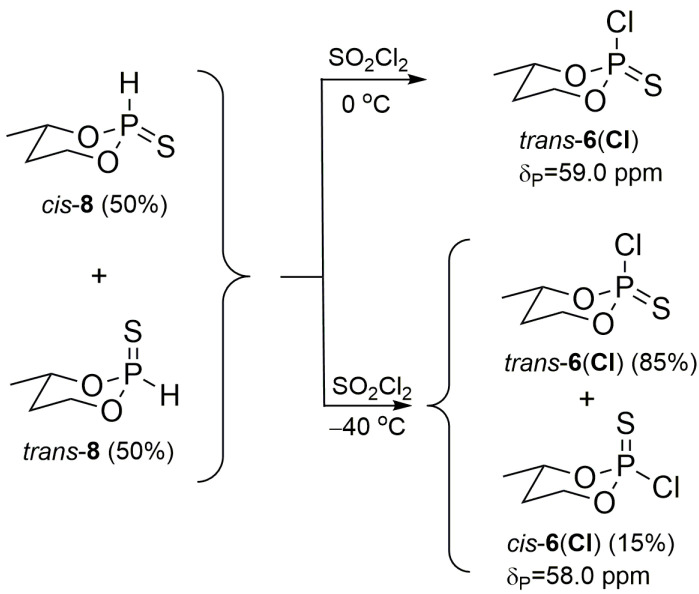
Stereospecific synthesis of *trans*-2-chloro-4-methyl-1,3,2-dioxaphosphorinan-2-thione **6**(**Cl**) from diastereomeric thiophosphites **8**.

**Scheme 5:**
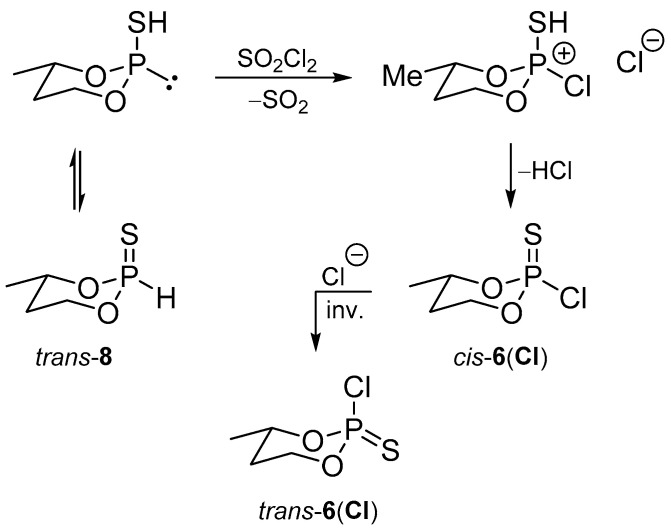
Proposed pathway of conversion of thiophosphite *trans*-**8** into thiophosphoryl chloride *trans*-**6**(**Cl**).

The results of the chlorination reaction of diastereomeric thiophosphites **8** summarized in Scheme 4 indicated that the diastereomer *trans*-**6**(**Cl**) is thermodynamically more stable than *cis*-**6**(**Cl**) and that epimerization at phosphorus was occurring in the reaction course. A possibility of epimerization of the starting thiophosphites **8** (*trans*-**8**→*cis*-**8**) was excluded since treatment of a 1:1 mixture of isomeric **8** with gaseous hydrogen chloride under the reaction conditions comparable to the chlorination reaction did not affect the isomeric ratio of **8**. Most probably, *cis*-**6**(**Cl**), when formed from *trans*-**8**, undergoes conversion to the more stable *trans*-**6**(**Cl**) via the chloride–chloride exchange reaction at phosphorus as shown below (Scheme 5).

**Scheme 6:**
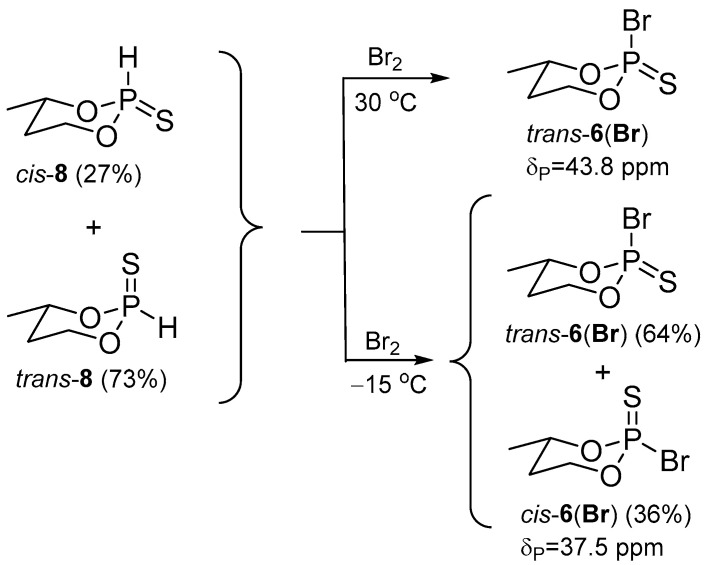
Stereospecific synthesis of *trans*-2-bromo-4-methyl-1,3,2-dioxaphosphorinan-2-thione **6**(**Br**).

The observation of a fully stereospecific formation of *trans*-**6**(**Cl**) upon chlorination of a mixture of isomeric thiophosphites **8** prompted us to investigate their bromination (Scheme 6). It was found that the reaction of thiophosphite **8** consisting of 73% *trans*-**8** and 27% *cis*-**8** with elemental bromine carried out at 30 °C in benzene gave only one crystalline isomer of phosphorobromidate trans-**6**(**Br**) in 85% yield. As in the case of *trans*-**6**(**Cl**), the C_4_-methyl protons of the crystalline isomer appeared in ^1^H-NMR spectrum as a double doublet with characteristic coupling constants, ^3^*J*_CH3-H_ = 6.9 Hz and ^4^*J*_CH3-P_ = 2.9 Hz, indicating that it exists in a chair conformation with both the C_4_-methyl and P=S groups in equatorial position. Moreover, Figure 4 shows that the shape of the resonance signal in the ^31^P-NMR spectrum of the more stable crystalline thiophosphoryl bromide *trans*-**6**(**Br**) is almost the same as that of the corresponding chloride *trans*-**6**(**Cl**). When the same mixture of isomeric thiophosphites **8** was reacted with bromine at −15°C, a mixture of both isomeric phosphorobromidates was formed, i.e., *trans*-**6**(**Br**) (64%) and *cis*-**6**(**Br**) (36%).

**Scheme 7:**
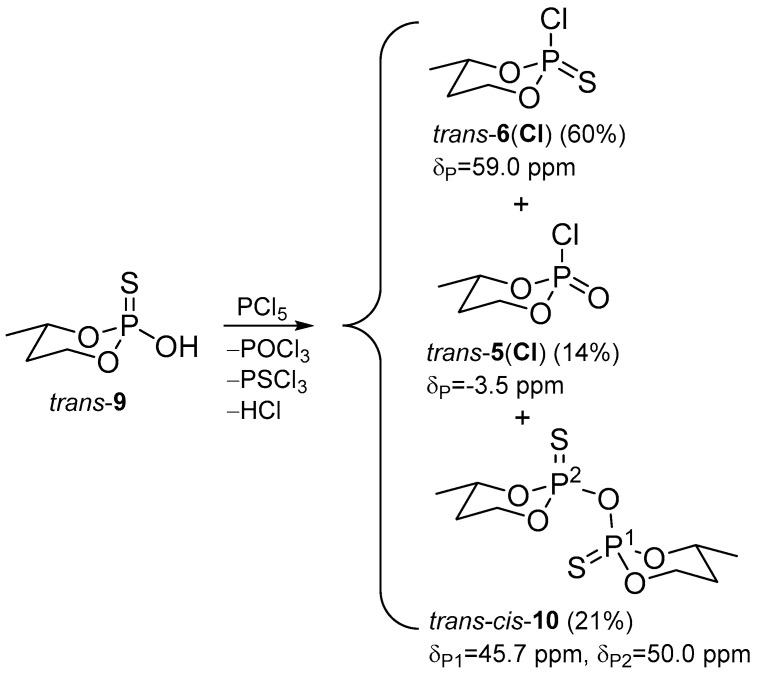
Structure of products formed in reaction between phosphorus pentachloride and thiophosphoric acid *trans*-**9**.

In search of a stereospecific synthesis of the less stable cyclic thiophosphoryl chloride *cis*-**6**(**Cl**), we turned our attention on the reaction of monothiophosphoric acids with phosphorus pentachloride. This reaction with the enantiomers of thiophosphonic acid **1** afforded exclusively enantiomerically pure thiophosphonyl chlorides **2** with inversion of configuration [2,4,22]. In the hope that cyclic monothiophosphoric acids will react with phosphorus pentachloride in a similar way, the reaction of easily available *cis*- and *trans*-2-hydroxy-4-methyl-1,3,2-dioxaphosphorinan-2-thiones **9** [23] withthis reagent was investigated. However, we were surprised to find that in addition to the expected isomeric chlorides **6**(**Cl**), two other products were formed. Their structures were dependent on the configuration at phosphorus in starting cyclic thioacids **9**. Thus, treatment of *trans*-**9** with PCl_5_ suspended in benzene at 0–5 °C resulted in formation of three products as revealed by ^31^P-NMR spectra: thiophosphoryl chloride (*trans*-**6**(**Cl**), phosphoryl chloride *trans*-**5**(**Cl**) and unsymmetrical *trans*-*cis*-dithiopyrophosphate **10** (see Scheme 7).

The formation of thiophosphoryl chloride *trans*-**6**(**Cl**) in this reaction was expected as well as the fact that it was formed from thioacid *trans*-**9** with inversion of configuration at phosphorus. However, the appearance of phosphorochloridate *trans*-**5**(**Cl**) among the reaction products was astonishing. Moreover, the replacement of sulfur in *trans*-**9** by chlorine leading to *trans*-**5**(**Cl**) was occurring with retention at phosphorus. The third product was identified as one of the three isomeric bis-(4-methyl-1,3,2-dioxaphosphorinan-2-thionyl)oxides **10**. Their synthesis, configuration and conformation were reported in a separate paper [24].

**Scheme 8:**
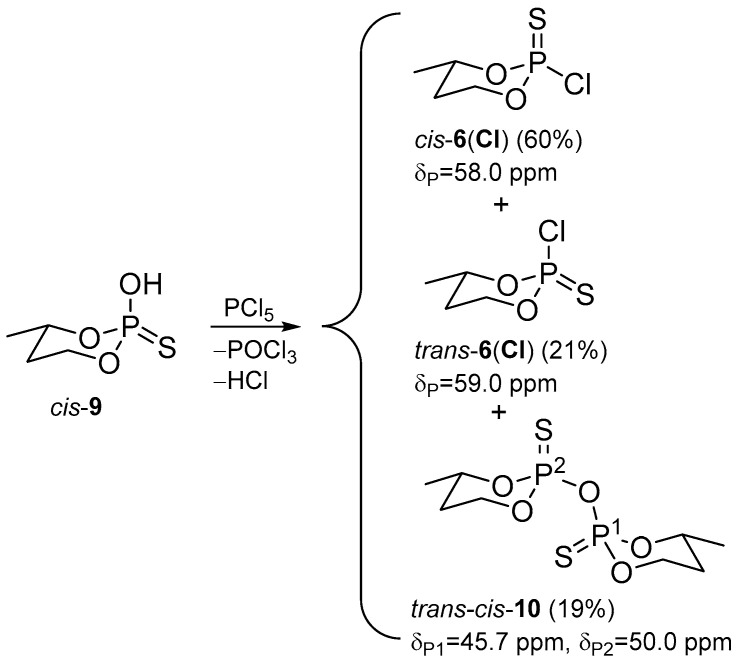
Structure of products formed in reaction between phosphorus pentachloride and thiophosphoric acid *cis*-**9**.

A somewhat different outcome of the reaction of thioacid *cis*-**9** with PCl_5_ was observed. To our satisfaction the major product in this case was the desired thermodynamically less stable thiophosphoryl chloride *cis*-**6**(**Cl**) formed in 60% yield. It was accompanied by the more stable isomer *trans*-**6**(**Cl**) (21%). The latter was undoubtedly the product of epimerization at phosphorus in *cis*-**6**(**Cl**) taking place in the presence of chloride anions in a reaction mixture. Interestingly, the same dithiopyrophosphate *trans*-*cis*-**10** was also formed in this reaction in comparable amounts (19%) (Scheme 8). Distillation of a mixture of these three reaction products allowed us to isolate thiophosphoryl chloride *cis*-**6**(**Cl**) containing 5% *trans*-isomer as the lowest boiling fraction. This almost diastereomerically pure *cis*-chloride **6** was used in our further experiments.

**Scheme 9:**
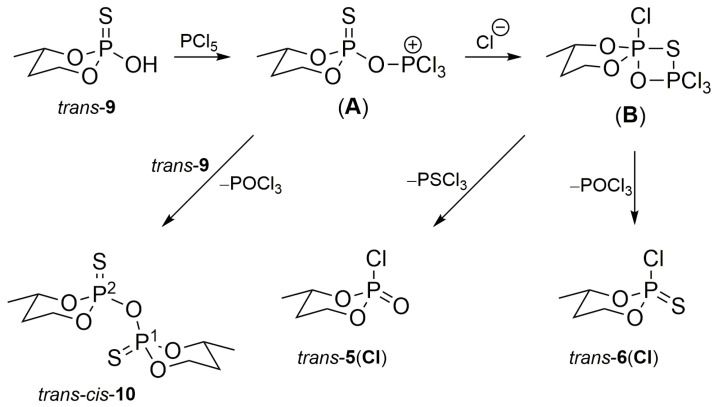
Proposed course of reaction between thioacid *trans*-**9** and phosphorus pentachloride.

Although elucidation of all mechanistic details of the reaction under discussion was beyond the scope of the present work, we wish to rationalize herein its multidirectional course and stereochemistry of the products formation (Scheme 9). Taking into account the well-known ambident reactivity of monothiophosphoric acids and their anions, it is reasonable to assume that cyclic thioacids, like acyclic ones, react with phosphorus pentachloride at the oxygen atom. This initial reaction, for example with *trans*-**9**, results in formation of *O*-trichlorophosphonium chloride (**A**) as the first reaction intermediate. This, in turn, upon a nucleophilic attack of the chloride anion at the phosphorinanyl phosphorus and the concomitant four-membered ring closure between sulfur and the trichlorophosphonium phosphorus atom, is converted into a second intermediate (**B**). Its formation and relative stability are due to the presence of two six- and four-membered rings, which are known to stabilize hypervalent phosphorus compounds. Interestingly, almost the same type of structure was postulated for the intermediate found in the reaction between oxophosphoranesulfenyl chlorides and phosphorus trichloride [25].

This trigonal bipyramidal intermediate (**B**) may undergo decomposition in two ways. The first accompanied by elimination of phosphoryl trichloride affords the expected thiophosphoryl chloride *trans*-**6**(**Cl**) with the experimentally confirmed inversion of configuration. On the other hand, simultaneous elimination of thiophosphoryl trichloride from (**B**) results in formation of the unexpected phosphoryl chloride *trans*-**5**(**Cl**) with retention of configuration at phosphorus. This stereochemistry of the conversion *trans*-**9→***trans*-**5**(**Cl**) is also in a full agreement with that experimentally found (see Scheme 9). Finally, the formation of dithiopyrophosphate **10** as a third reaction product is worthy of notice. It is undoubtedly formed in the reaction between starting thioacid *trans*-**9** and the first reaction intermediate (**A**). The latter, having a good leaving group, undergoes nucleophilic substitution by thioacid *trans*-**9** with inversion of configuration at phosphorus, which affords unsymmetrical *trans*-*cis*-diastereomer of dithiopyrophosphate **10**.

**Scheme 10:**
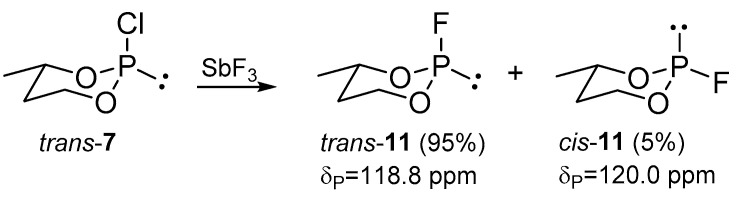
Synthesis of fluorophosphite **11**.

To complete the synthesis of cyclic thiophosphoryl halogenides **6**(**Cl**, **Br**, **F**) and having some experience with the preparation of chlorides **6**(**Cl**) and bromides **6**(**Br**), we turned to the synthesis of diastereomeric fluorides **6**(**F**). In general, the synthetic approaches to acyclic and cyclic thiophosphoryl fluorides are sparse and of limited applicability. The most practical method of their synthesis involves the halogen exchange reaction at phosphorus using inorganic fluorides [3,26]. In our first attempt to obtain **6**(**F**), a very simple reaction was tested, namely, the addition of elemental sulfur to cyclic fluorophosphite **11**. The latter was prepared according to the modified procedure described by Schmutzler [27] from *trans*-chlorophosphite **7** and antimony trifluoride (Scheme 10).

**Scheme 11:**
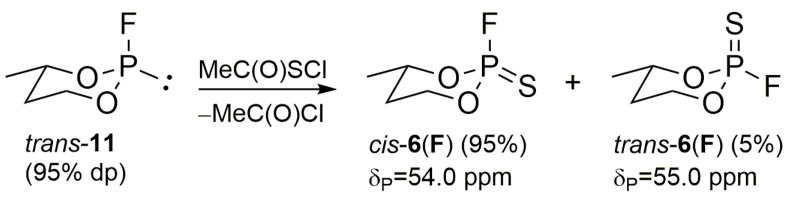
Synthesis of thiophosphoryl fluoride **6**(**F**).

*Trans*-fluorophosphite **11** as a major and thermodynamically more stable product obtained in this reaction was contaminated with 5% *cis*-isomer. To our surprise, it was found that the addition of elemental sulfur to *trans*-**11** does not occur even in boiling toluene. When, however, acetylsulfenyl chloride was used instead of elemental sulfur, the conversion of *trans*-**11** to *cis*-**6**(**F**) was achieved efficiently under mild reaction conditions (Scheme 11).

**Figure 5:**
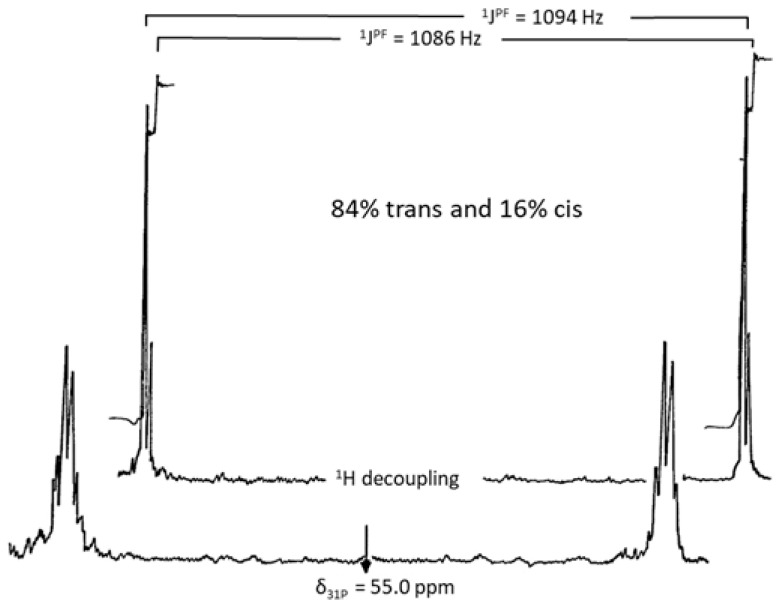
^31^P-NMR spectra of *trans*-**6**(**F**) (85%) and *cis*-**6**(**F**) (15%) mixture: upper spectrum with a proton decoupling, bottom spectrum without proton decoupling.

As expected, the sulfurization of *trans*-fluorophosphite **11** occurred with a full retention of configuration at phosphorus and afforded the crude thiophosphoryl fluoride *cis*-**6**(**F**) containing 5% of *trans*-**6**(**F**). The diastereomerically pure solid *cis*-isomer (m.p. 33–34 °C) was isolated from this mixture by distillation and crystallization. The NMR (^1^H, ^31^P) data of *cis*-fluoride **6**(**F**) were consistent with a rigid chair conformation in which the thiophosphoryl sulfur and the methyl group on C_4_ are in equatorial positions on the 1,3,2-dioxaphosphorinan ring. For instance, the resonance signal (double doublet) of the methyl protons with characteristic coupling constants, ^3^*J*_CH3-H_ = 6.2 Hz and ^4^*J*_CH3-P_ = 2.6 Hz, shows that the methyl group is equatorial. The ^31^P-NMR spectrum of *cis*-**6**(**F**) shows a typical doublet with the coupling constant ^1^*J*_P-F_= 1086 Hz due to the presence of fluorine directly bonded to phosphorus. The shape of each arm of this doublet is almost identical with the ^31^P-NMR resonance signals of *trans*-**6**(**Cl**) and *trans*-**6**(**Br**) (see Figure 1). This observation may be taken as additional evidence that all the thiophosphoryl halogenides mentioned above exist in the same chair conformation with the equatorial C_4_-methyl and thiophosphoryl group. Finally, a single-crystal X-ray analysis of *cis*-**6**(**F**) revealed [28] that the 1,3,2-dioxaphosphorinan ring also adopts a chair conformation in the solid state and confirmed our configurational assignments based on NMR measurements (see Figure 2 and Figure 5).

**Scheme 12:**
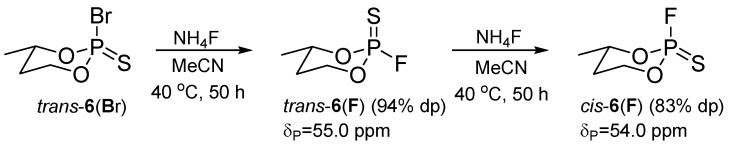
Synthesis of thiophosphoryl fluoride *trans*-**6**(**F**) and its epimerization to *cis*-**6**(**F**).

The *trans*-isomer of **6**(**F**) was obtained from *trans*-bromide **6**(**Br**) and ammonium fluoride. The bromide → fluoride exchange reaction carried out in acetonitrile solution at 40 °C afforded after 8 h a mixture of *trans*- and *cis*-fluorides at a ratio of 84:16. When the reaction was conducted for 24 h at the same temperature, the desired *trans*-fluoride **6**(**F**) was formed with inversion of configuration and with 94% diastereomeric purity. The diastereomerically pure *trans*-**6**(**F**) was obtained by preparative gas chromatography. However, prolongation of the reaction time to 50 h resulted in the isomerization of *trans*-**6**(**F**) (inversion of configuration at P) to the thermodynamically more stable *cis*-**6**(**F**) and the latter was formed with 83% diastereomeric purity (Scheme 12).

**Scheme 13:**
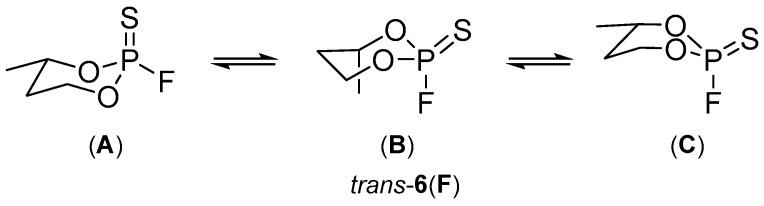
Conformational equilibrium in *trans*-**6**(**F**).

Most probably, the above fluoride–fluoride exchange reaction should lead to the diastereomerically pure *cis*-**6**(**F**) after longer heating. Moreover, the fact that a trace of the less stable *trans*-**6**(**F**) was not observed when the pure fluoride *cis*-**6**(**F**) was heated with NH_4_F (60 h, 40 °C) indicates that this identity reaction reflects a strong preference of fluorine for an axial position versus a P=S bond in the 1,3,2-dioxaphosphorinan ring. DFT calculations show that the *trans*-**6**(**F**) isomer has a free energy of ΔG = 2.5 kcal/mol higher than *cis*-**6**(**F**) isomer, which corresponds roughly to an equilibrium concentration ratio [*trans*-**6**(**F**)]_eq_/[*cis*-**6**(**F**)]_eq_ of ca. 1.5 × 10^−2^. Taking into account ^1^H- and ^31^P-NMR data, as well as DFT calculations (see Supplementary Materials), it is reasonable to assume that the *trans*-**6**(**F**) isomer exists in two chair conformations (**A** and **B**) and one boat conformation(**C**) that are in a fast equilibrium as shown below (Scheme 13).

The conformer (**B**) with the axial methyl group at C_4_ should be present in equilibrium in substantial proportions, which is reflected in the different shape of the diagnostic ^1^H-NMR resonance signal of the C_4_-Me from that of the *cis*-isomer **6**(**F**). Thus, the methyl protons of *trans*-**6**(**F**) appeared in the spectrum as a doublet of triplets due to characteristic splitting by the C_4_-proton (^3^*J*_CH3-H_ = 6.5 Hz), then by phosphorus (^4^*J*_CH3-P_ = 0.9 Hz) and additionally by the vicinal axial proton at C_5_ with the same coupling constant value (0.9 Hz) as with phosphorus. The observation of the last coupling and a small value of the coupling constant with phosphorus is indicative of the presence of the conformer (**B**) in equilibrium. On the other hand, the ^31^P-NMR spectrum of *trans*-**6**(**F**) shows a typical doublet due to splitting by fluorine with the coupling constant ^1^*J*_P-F_ = 1094 Hz. Both arms of this doublet are dense multiplets, which are characteristic of the dioxaphosphorinan rings, having methyl and thiophosphoryl groups *trans* situated. The ^31^P-NMR spectra of a mixture of *trans*-**6**(**F**) (84%) and *cis*-**6**(**F**) (16%) (shown in Figure 5) in conjunction with Figure 4 best illustrate the relationship between the shape of the ^31^P-NMR resonance signals and configuration at phosphorus and C_4_-carbon in the 1,3,2-dioxaphosphorinan systems.

These observations are supported by our DFT calculations. We performed full conformational analysis of one pair of diastereomers, namely (*R_P_,S_C4_*) and (*S_P_,S_C4_*), as the other pair, (*S_P_,R_C4_*) and (*R_P_,R_C4_*), respectively, are their equivalent enantiomeric forms. For both diastereomers, two stable low energy chair conformations and one boat conformation were identified. The structures and relative free energies of the *cis*- and *trans*-conformers of **6**(**F**) in aqueous solution (in kcal/mol) are given in Supplementary Materials, Figure S2 and Table S2. The calculated geometry of *cis*-2-fluoro-4-methyl-1,3,2-dioxaphosphorinan-2-thione in the aqueous solution agrees very well with its crystal structure reported [24], which proves the reliability of the theoretical method used (Supplementary Materials, Table S4).

The most stable conformer of the two diastereomers (Scheme 11) is the conformer *cis*-**6**(**F**) in which fluorine occupies an axial position and the methyl group on the C_4_ carbon occupies an equatorial position.

### 2.2. Stereochemistry of Nucleophilic Displacement Reactions at Phosphorus in 2-Halogeno-4-methyl-1,3,2-dioxaphosphorinan-2-thiones **6**

Having in hand the diastereomeric pairs of thiophosphoryl chloride **6**(**Cl**) and fluoride **6**(**F**), we could start with investigation of the stereochemical course of nucleophilic displacement processes occurring at phosphorus. In the present work, three basic substitution reactions were examined, namely alkaline hydrolysis, methanolysis and aminolysis. The choice of the first two reactions was due to the fact that they produce diastereomeric thioacids **9 [23]** and thionophosphates **12 [19]**, respectively, which were obtained by us in an independent way and the configuration at phosphorus in both diastereomers of **9** and **12** was firmly established. Moreover, the diastereomeric hydrolysis products **9** as well as thiophosphoryl amides **13** formed in the third reaction do not undergo epimerization at phosphorus under the reaction conditions.

**Scheme 14:**
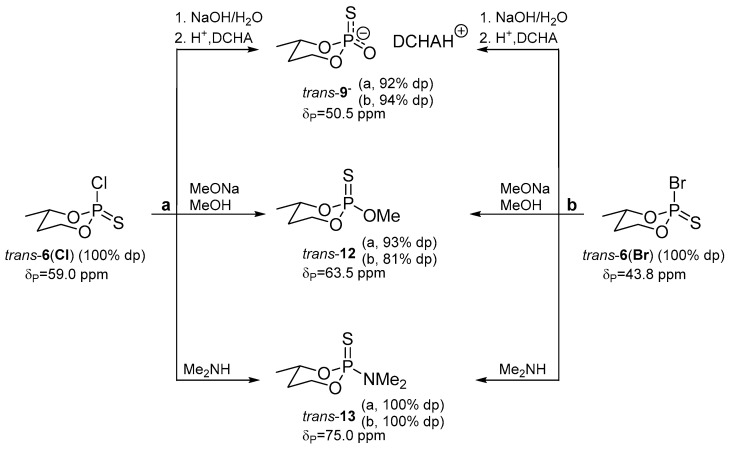
Nucleophilic displacement reactions at phosphorus in cyclic thiophosphoryl chloride *trans*-**6**(**Cl**), path **a**, and bromide *trans*-**6**(**Br**), path **b**.

At first, the stereochemical course of displacement reactions at phosphorus in the *trans*-isomer of thiophosphoryl chloride **6**(**Cl**) was investigated. The results obtained are summarized in Scheme 14.

**Scheme 15:**
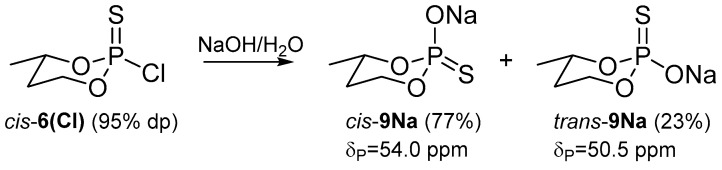
Alkaline hydrolysis of thiophosphoryl chloride *cis*-**6**(**Cl**).

Alkaline hydrolysis of the diastereomerically pure *trans*-**6**(**Cl**) as well as other halogenides **6** was carried out in a water-dioxane solution at ca. 0 °C. The crude sodium salt of thioacid **9** isolated from the reaction mixture showed two resonance signals at δ_P_ = 50.5 and 54.0 ppm in the ^31^P-NMR spectrum, corresponding to the sodium salts of thioacid *trans*-**9** and *cis*-**9,** respectively. They were formed in a 92:8 ratio. For further identification and characterization, this mixture of both sodium salts was converted into the corresponding dicyclohexyl ammonium salts of *trans*-**9** (92%) and *cis*-**9** (8%) displaying chemical shifts at δ_P_ = 50.5 and 53.5 ppm. The fact that thioacid *trans*-**9** was formed as the major hydrolysis product indicates that the reaction under discussion occurred with inversion of configuration at phosphorus. A very small amount (8%) of thioacid *cis*-**9** formed in this reaction may be explained by a concomitant formation of *cis*-**6**(**Cl**) (via the chloride–chloride exchange reaction in the starting *trans*-**6**(**Cl**)) and its subsequent hydrolysis to *cis*-**9**. Alkaline hydrolysis of the thermodynamically less stable thiophosphoryl chloride *cis*-**6**(**Cl**) with 95% diastereomeric purity afforded a mixture of two sodium salts of thioacid *cis*-**9** and *trans*-**9** in a 77:23 ratio as revealed by ^31^P-NMR spectrum. Formation of the sodium salt of *cis*-**9** as the major product also demonstrated the prevailing stereoinvertive course of hydrolysis of this stereomer (Scheme 15).

The methanolysis reaction of *trans*-**6**(**Cl**) was carried out in a mixture of benzene and sodium methoxide in methanol at 0 °C. It afforded the cyclic thionophosphate *trans*-**12** with 93% diastereomeric purity (^31^P-NMR assay). As in the case of alkaline hydrolysis of *trans*-**6**(**Cl**), the stereochemical outcome of the methanolysis was also inversion of configuration at phosphorus. However, in this case a small decrease of diastereoselectivity in this reaction may be due to the methoxy–methoxy exchange reaction at phosphorus in *trans*-**12**, resulting in formation of *cis*-**12**, the more thermodynamically stable isomer.

**Scheme 16:**
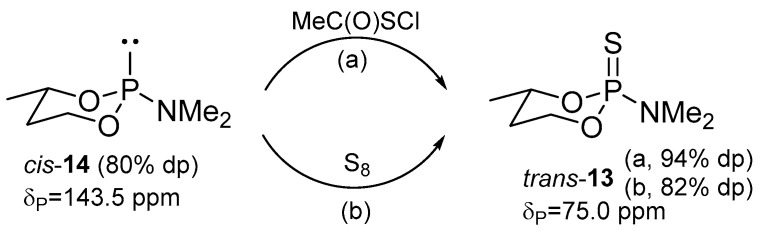
Sulfurization of cyclic amidophosphite *cis*-**14** with MeC(O)SCl (**a**) and S_8_ (**b**).

In contrast to alkaline hydrolysis and methanolysis, the reaction of *trans*-**6**(**Cl**) with dimethylamine in ether at 0 °C gave the corresponding thiophosphoryl amide *trans*-**13** with complete inversion of configuration at phosphorus. As the aminolysis product (*trans*-**13**) was not reported in the literature, it was prepared by us in an independent way starting from *cis*-2-dimethylamino-4-methyl-1,3,2-dioxaphosphorinan **14** (80% dp) [29], which upon treatment with acetylsulfenyl chloride gave thiophosphoryl amide *trans*-**13** with 94% diastereomeric purity. Addition of elemental sulfur to amidophosphite **14** also occurred with retention of configuration at phosphorus and afforded *trans*-**13** with 82% diastereomeric purity (Scheme 16).

Similar results were obtained with the thiophosphoryl bromide *trans*-**6**(**Br**), which was used as a substrate in the three substitution reactions investigated herein (see Scheme 14). Thus, alkaline hydrolysis and methanolysis of this bromide were found to occur with a predominant inversion of configuration at phosphorus while aminolysis, as in the case of the *trans*-chloride **6**(**Cl**), was fully diastereoselective and gave the pure *trans*-**13** with inversion of configuration. In this connection, it is interesting to mention that the condensation reactions of isomeric *cis*- and *trans*-chlorides and bromides, **6**(**Cl**) and **6**(**Br**) with the diastereomeric thioacid **9** anions occurred almost exclusively by inversion of P-configuration [24].

**Scheme 17:**
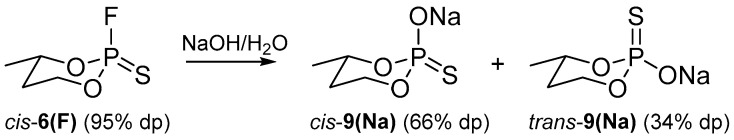
Alkaline hydrolysis of thiophosphoryl fluoride *cis*-**6**(**F**).

Before discussing our present results on displacement reactions in the diastereomeric thiophosphoryl fluorides **6**(**F**), it is necessary to note that Inch and coworkers [30] reported very early that the displacement of fluorine by nucleophilic reagents in the 1,3,2-dioxaphosphorinan-2-ones derived from carbohydrates occurs with preponderant retention at phosphorus. However, the stereochemical course of these reactions was examined with only one, more stable diastereomer with the axial fluorine. Therefore, the discussion on the stereochemistry-mechanism relationship was rather limited and not conclusive. As we have been successful in preparing and purifying both *cis*- and *trans*-fluorides **6**(**F**), we decided to concentrate our attention on their alkaline hydrolysis. In the first place, this reaction was investigated with the thermodynamically more stable thiophosphoryl fluoride *cis*-**6**(**F**), and also with the axial fluorine, to compare our results with those reported by Inch. Thus, hydrolysis was carried out according to a general procedure also applied for other thiophosphoryl halogenides **6** (NaOH, H_2_O-dioxane, 0 °C) and resulted in a mixture of the two sodium salts of the thioacid *cis*-**9** and *trans*-**9** in a ratio of 66:34 as estimated by ^31^P-NMR spectra [27] (see Scheme 17).

This result clearly showed that the fluoride *cis*-**6**(**F**) underwent hydrolytic conversion to *cis*-**9(Na)** as the major product with retention of configuration at phosphorus. In this way, the results of Inch were to some extent corroborated. However, the most intriguing was a high content of the sodium salt of *trans*-**9,** which was simultaneously formed in this reaction with inversion of configuration, as epimerization of the more stable *cis*-**6**(**F**) to its less stable *trans*-isomer does not occur in the presence of fluoride anion. Such a stereochemical outcome of alkaline hydrolysis should be due to a different mechanism of hydrolysis of 2-fluorophosphorinanes **6**(**F**) as compared with their chloro- and bromo-analogues. We have also noted a negligible effect of the reaction medium and added inorganic salts on the retention-inversion ratio (see Table 2).

**Scheme 18:**
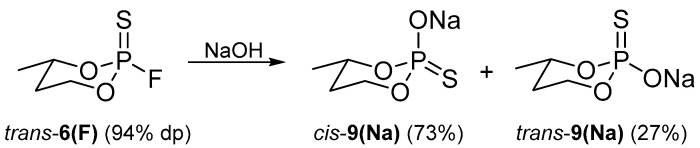
Alkaline hydrolysis of thiophosphoryl fluoride *trans*-**6**(**F**).

The results of alkaline hydrolysis of the less stable fluoride *trans*-**6**(**F**) (equatorial fluorine) were even more interesting. In this case, two sodium salts of the thioacid **9** were obtained in a 73:27 ratio; however, the major product, *cis*-**9**(**Na**), was formed with inversion of configuration (see Scheme 18).

In additional experiments, it was found that diastereomeric purity of the starting thiophosphoryl fluoride *trans*-**6**(**F**) has practically no effect on the product ratio, which is in this case equivalent to the inversion-retention ratio (see Table 3).

**Scheme 19:**
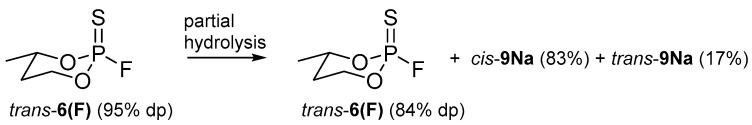
Partial alkaline hydrolysis of thiophosphoryl fluoride *trans*-**6**(**F**).

To estimate the effect of epimerization of the starting *trans*-fluoride **6**(**F**) to *cis*-isomer by fluoride ion, alkaline hydrolysis was performed with an insufficient molar amount of sodium hydroxide. The results obtained are shown in Scheme 19. It turned out that the unreacted fluoride *trans*-**6**(**F**) was only slightly epimerized and recovered with 84% diastereomeric purity. Therefore, this side process considered here cannot essentially change the main stereochemical outcome of hydrolysis.

**Scheme 20:**
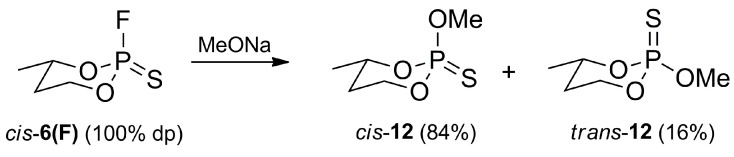
Methanolysis of thiophosphoryl fluoride *trans*-**6**(**F**).

Finally, for comparison purposes, the reaction of the more stable thiophosphoryl fluoride *cis*-**6**(**F**) with sodium methoxide was briefly investigated. In accord with the results of alkaline hydrolysis of this thiofluoridate, its methanolysis afforded both isomeric thionophosphates*cis*-**12** and *trans*-**12**, the former being the major product was formed with retention of configuration as shown in Scheme 20.

It was also found that the product isomer ratios are practically not influenced by solvents, both protic and aprotic, indicating that they reflect the mode of substitution (Table 4).

Dissecting the results of our studies on the stereochemistry of displacement reactions at phosphorus in 2-halogeno-4-methyl-1,3,2-dioxaphosphorinan-2-thiones **6** presented above several important observations and interpretations can be inferred. First of all, there is a sharp difference in the stereochemical course of substitution reactions between diastereomeric thiophosphoryl chlorides and bromides **6**(**Cl**, **Br**) on the one side and fluorides **6**(**F**) on the other side. Both diastereomeric chlorides **6**(**Cl**) and bromides **6**(**Br**) react with nucleophilic reagents as a rule with full inversion of configuration at phosphorus. A small decrease of diastereoselectivity observed in alkaline hydrolysis and methanolysis is due to concomitant side epimerization reactions of a substrate and/or a product of the displacement reaction investigated. In the case of thiophosphoryl chlorides **6**(**Cl**) and bromides **6**(**Br**), the substitutions are kinetically controlled, which implies that inversion at phosphorus may be a consequence of a direct S_N_2-P type displacement.

In contrast to the diastereomeric chlorides **6**(**Cl**), both diastereomeric fluorides (*cis*-**6**(**F**) and *trans*-**6**(**F**)) undergo alkaline hydrolysis, affording the same mixture of the corresponding thioacids **9** with the thermodynamically more stable thioacid *cis*-**9** being a major product in both cases. It means that hydrolysis of *cis*-**6**(**F**) affords *cis*-**9** with retention of configuration, while *trans*-**6**(**F**) upon hydrolysis gives the same major product with inversion of configuration. Such a stereochemical outcome of alkaline hydrolysis of both isomeric fluorides **6**(**F**) and other observations presented above indicate that this reaction as well as methanolysis are controlled by thermodynamic factors. Taking into account well known facts that (a) fluorine forms a very strong bond with phosphorus, (b) the fluoride anion is a poor leaving group, and (c) fluorine as a substituent stabilizes pentacoordinate phosphorus and is strongly apicophilic, it is believed that nucleophilic substitution at phosphorus in thiophosphoryl fluorides **6**(**F**) occurs according to the addition–elimination (A–E) mechanism.

### 2.3. DFT Studies on Mechanism-Stereochemistry Relationship inSubstitution of Cyclic 2-halogeno-4-methyl-1,3,2-dioxaphosphorinane-2-thiones **6**

The conformation and structure of 1,3,2-dioxaphosphorinanes have been extensively studied by ^1^H-, ^13^C- and ^31^P-NMR, IR, and X-ray spectroscopies [10,31]. Although theoretical studies of various 1,3,2-dioxaphosphorinanes have also been published [32,33,34], to the best of our knowledge, the hydrolysis reaction of 2-halogeno-4-methyl-1,3,2-dioxaphosphorinan-2-thiones **6** was not investigated by theoretical methods. Therefore, having in mind the results of our stereochemical studies and suggestions concerning the different mechanisms of the alkaline hydrolysis of both cyclic diastereomeric thiophosphoryl chlorides **6**(**Cl**) and fluorides **6**(**F**), we decided to concentrate on elucidation stereochemical and mechanistic details of this reaction.

To calculate the energy of this substitution reaction, we first performed conformational analysis of reagents (**6**(**Cl**), **6**(**F**)) and the product, 2-hydroxy-4-methyl-1,3,2-dioxaphosphorinan-2-thione **9**. In the case discussed here, the presence of the methyl group at C_4_ in the six-membered ring induces stereoisomerism in the molecule, which may result in a different reactivity of diastereomers towards the nucleophile. Therefore, we calculated chair and boat conformations for both *cis*- and *trans*- diastereomers of chlorides **6**(**Cl**) and fluorides **6**(**F**) and compared the Gibbs free energies of all stable minima.

The most stable diastereomer of **6**(**Cl**) was found to exist in a chair conformation with chlorine in the axial position and methyl in the equatorial position, denoted in this work as *trans*-**6**(**Cl**). The remaining conformers are ΔG_rel_ = 2.5–5.6 kcal/mol higher in energy than *trans*-**6**(**Cl**). The relative Gibbs free energies of all isomers are collected in Table S1. These data show a strong preference for chlorine to the axial position. All isomers with chlorine occupying the equatorial position have much higher energies. Additional stabilization of *trans*-**6**(**Cl**) is due to the methyl group in equatorial arrangement, which minimizes the steric congestion. These observations support the common knowledge about these ring systems [10].

The stability order of conformers of **6**(**F**) is very similar in terms of relative stereochemistry. One should remember that due to the different priority of substituents, upon replacement of Cl for F, the most stable conformation is again that with axial fluorine and an equatorial methyl group, i.e., *cis*-**6**(**F**) (see Supplementary Materials, Table S2 and Figure S2). Comparison of the calculated structure of this isomer in the aqueous solution agrees very well with the reported crystal structure [28], which proves the reliability of the theoretical method used (Supplementary Materials, Table S4).

The most stable conformer of **9** is that with the axial hydroxyl and equatorial C_4_-methyl group, i.e., *cis*-**9**, as in the case of fluorine and chlorine. However, only slightly less stable (by 0.7 kcal/mol) is the isomer *trans*-**9** with both hydroxyl and methyl groups in equatorial positions (Supplementary Materials, Table S3 and Figure S3).

**Figure 6:**
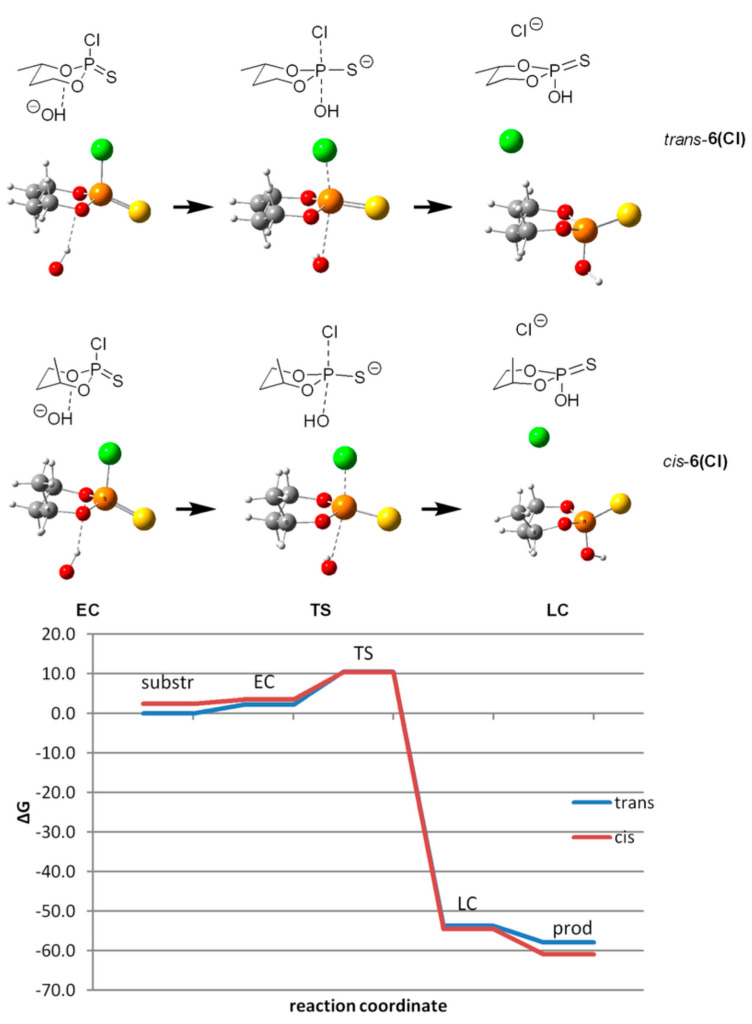
Relative free energy profile (kcal/mol) for the chloride–hydroxide exchange in *trans-* (blue line) and *cis-* (red line) **6(Cl)**; (**EC**)-early complex (complex of substrates), (**TS**)-transition state, (**LC**)-late complex (complex of products).

DFT calculations of the alkaline hydrolysis of *trans*-**6**(**Cl**) in water showed that indeed the preferred reaction route is the attack of hydroxide ion from the opposite side to chlorine and the ligand exchange occurs with inversion of configuration. It is a one-step reaction that proceeds via a single transition state as was found for acyclic alkoxyphosphoryl chlorides [35,36]. Both *trans* and *cis* isomers react according to the same S_N_2-P mechanism and their free energy profiles are almost identical (Figure 6).

**Scheme 21:**
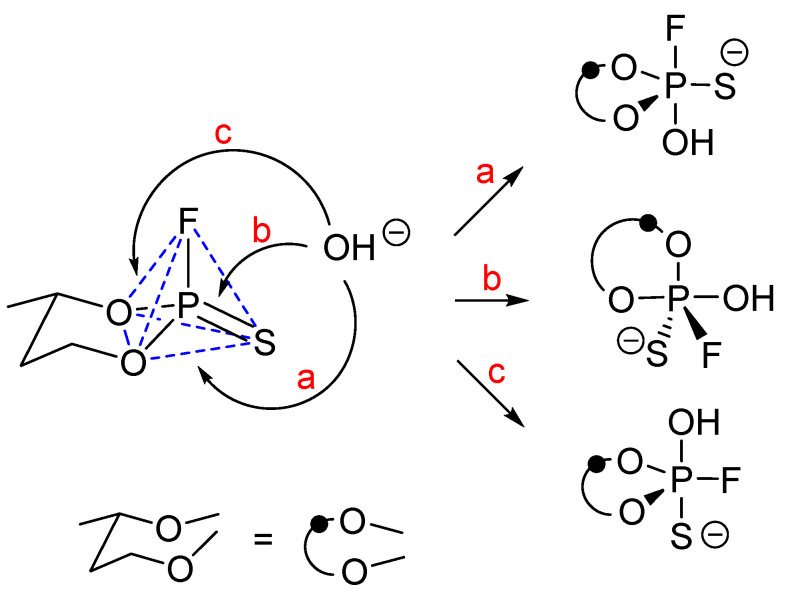
Three directions of the nucleophilic attack of hydroxide ion on phosphorus: (**a**) opposite to F, (**b**) opposite to oxygen in the ring (two variants possible as due to substitution at C_4_, the oxygens are slightly nonequivalent), (**c**) opposite to S.

The analogous hydrolysis of **6**(**F**) seems more complicated in view of the experimental results. To deeper understand the mechanistic and stereochemical details of this process, we performed analogous DFT calculations of the replacement of fluoride ion in *cis*- and *trans*-**6**(**F**) by hydroxyl anion in water solution. Anticipating that the reaction may occur stepwise according to the addition–elimination scheme [35], we considered at first the reaction pathways starting from the approach of the nucleophile to each face of the tetrahedron formed by S, O(C_4_), O(C_6_), and F (Scheme 21). The stationary points on the reaction pathway (intermediates and transition states) were identified by computing potential energy scans (PES) of the reaction. The route (**a**) should lead to formation of a trigonal bipyramidal intermediate (TBPI) with OH and F in apical positions. The route (**b**) consists of two directions of nucleophilic attack, from the opposite side relative to each oxygen in the ring. Energetically, both routes (**b-1** and **b-2**) are almost equivalent. The route (**c**) involves the attack of OH^−^ on phosphorus from the opposite side to sulfur (Scheme 21).

**Figure 7:**
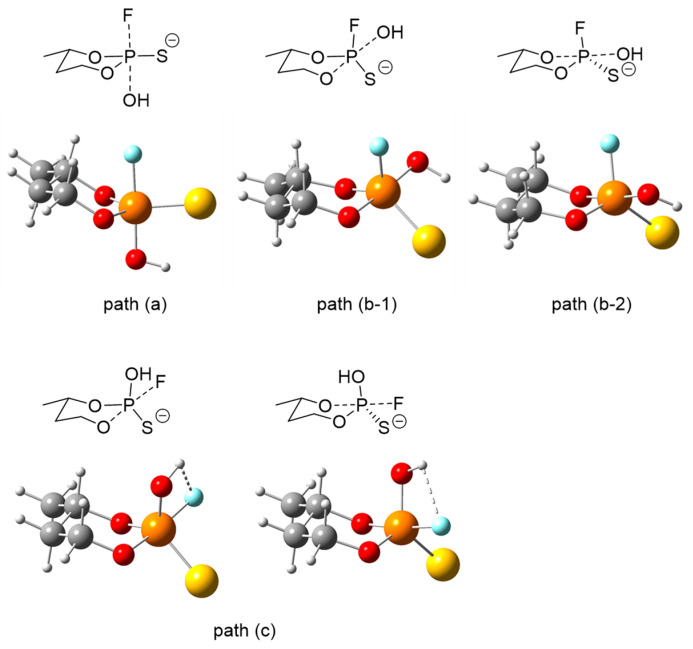
Structures of the P^V^ intermediates resulting from attack of OH^−^ on *cis*-**6**(**F**); for description of routes (**a**–**c**) see Scheme 21.

**Figure 8:**
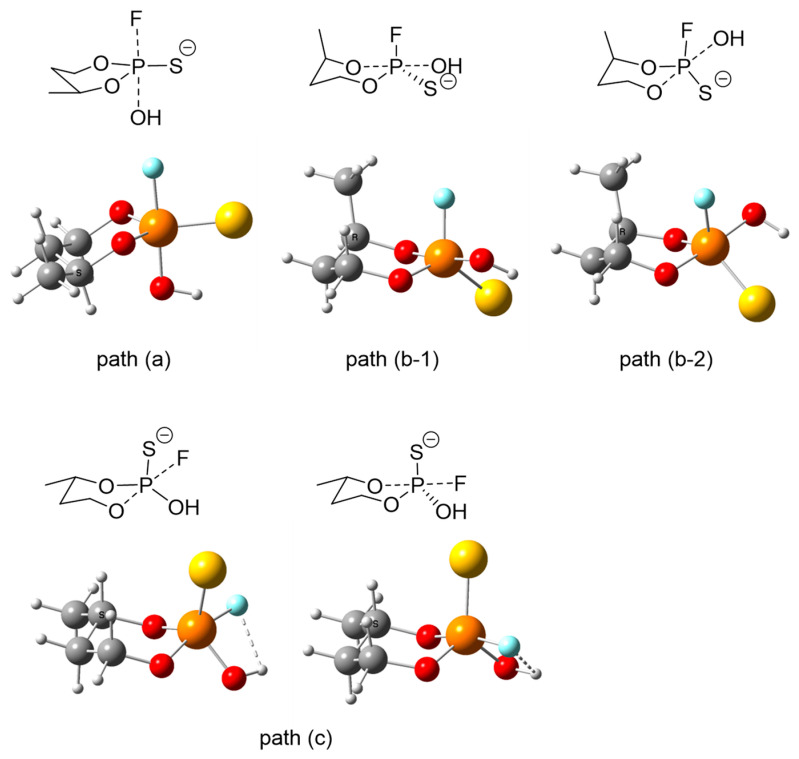
Structures of the P^V^ intermediates resulting from attack of OH^−^ on *trans*-**6**(**F**); for description of routes (**a**–**c**) see Scheme 21.

It should be mentioned that the frontside attack, although usually associated with a much higher energy barrier than the backside attack [37], is in this case possible as it leads to the TBPI with electronegative oxygen in the apical position. Moreover, the ring in such TBPI occupies the apical-equatorial position, which is the preferred arrangement in terms of the ring strain. In contrast to chloride substitution, it was found that all reaction routes involving fluoride **6**(**F**) proceed via pentacoordinate intermediates, as suggested by stereochemical studies (taking into account the high electronegativity and apicophilicity of fluorine). Below, all P^V^ intermediate structures identified for reaction pathways (**a**)–(**c**) of *cis* and *trans* diastereomers of **6(F)** are presented in Figure 7 and Figure 8).

The lowest free energy barrier in the case of *cis*-**6**(**F**) is associated with the classical backside attack of hydroxyl relative to fluorine (path **a**). The side attack (**b**), which places the ring in apical-equatorial position, has the barrier by ca. 1 kcal/mol higher (Table 5). The approach of OH^−^ from the back direction relative to sulfur is associated with the highest free energy barrier (by 8 kcal/mol higher than (**a**)), so it is the least likely. However, calculations showed that this pathway leads to the most stable TBPI, which also loses fluoride most readily to give the thioacid **9**, as can be seen in Supplementary Materials, Figure S7. Thus, from the kinetic point of view, the formation of intermediate (**a**) seems preferable. Most probably, the pentacoordinate intermediate formed as a result of backside attack (**a**) can undergo a sequence of reversible pseudorotations, eventually leading to a major product with retention of configuration on P, in accord with thermodynamic equilibrium [38]. Indeed, we have identified at least one sequence of pseudorotations linking both intermediates **6**(**F**)-**a** and **6**(**F**)-**c** (Scheme 14).

**Figure 9:**
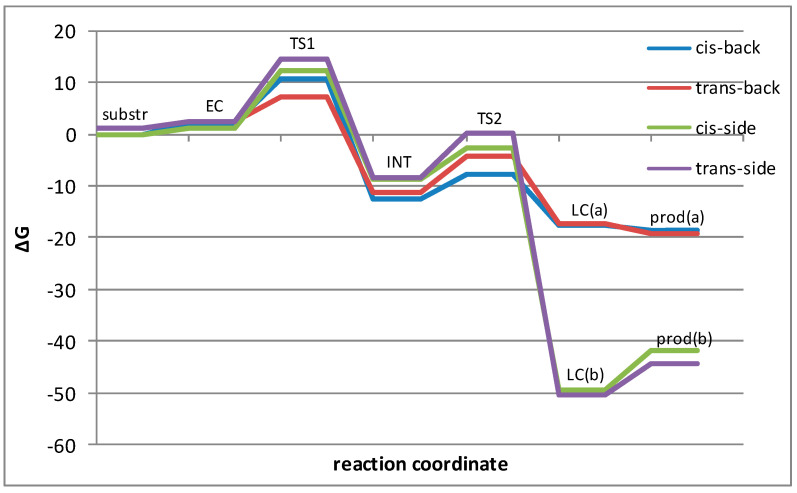
Free energy profiles for the substitution of fluorine by OH^−^ in *cis* and *trans* diastereomers of **6(F)**; attack from the back to the fluorine (back) and from the back to oxygen in the ring (side); EC-complex of substrates (early complex), TS1-1st transition state (approach of OH^−^), INT-P^V^ intermediate, TS2-2nd transition state (departure of F^−^), LC-complex of products (late complex) according to route (**a**) or (**b**) and (**c**) (Scheme 21), respectively (Table 5 and Table 6).

The reactivity of the diastereomer *trans*-**6**(**F**) is somewhat different. The attack of OH^−^ from the opposite side to fluoride (**a**) has the lowest free energy barrier (by 7 kcal/mol lower than the attack (**b**)). The attack from the opposite side to sulfur (**c**) is again the most unfavourable. This suggests that the reaction with inversion of configuration should kinetically dominate, which is in accord with observations. The thermodynamic parameters of hydrolysis of both diastereomers are presented in Table 5 and Table 6 and the free energy profiles for routes (**a**) and (**b**) of *cis-* and *trans*-**6**(**F**) hydrolysis are compared in Figure 9. The difference in energies of the free products comes from the fact that in route (**a**) the free fluoride anion is formally produced as the byproduct while in routes (**b**) and (**c**) it is HF and thiophosphate anion. The reaction leading to hydrogen fluoride and thiophosphate anion is much more favourable thermodynamically but in alkaline medium; this of course is meaningless as the fast equilibrium between these species is immediately established.

Route (**a**): **>P(S)F + OH^(−)^ → >P(S)OH + F^(−)^**, ΔG = −18.6 (*cis*); −19.2 kcal/mol (*trans*).

Route (**b**) and (**c**): **>P(S)F + OH^(−)^ → >P(S)O^(−)^ + HF**, ΔG = −41.8 (*cis*); −44.4 kcal/mol (*trans*).

**Scheme 22:**
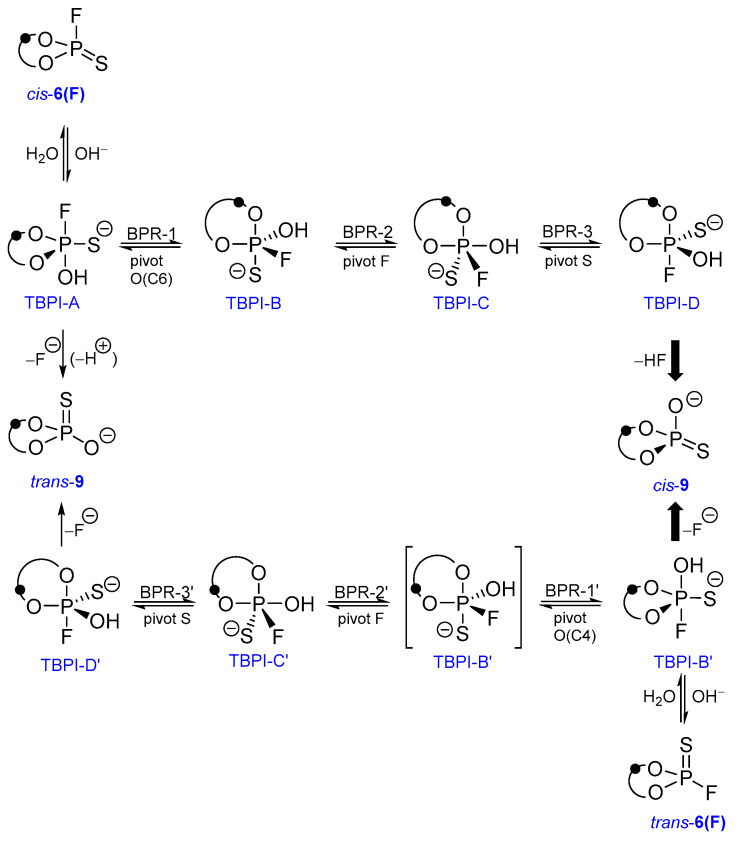
Mechanism of alkaline hydrolysis of *cis*-**6**(**F**) and *trans*-**6(F)** (bold arrows show formation of major product *cis*-**9** from *cis* and *trans* fluorides).

The DFT calculations presented above provided convincing evidence that alkaline hydrolysis of cyclic thiophosphoryl fluorides **6**(**F**) proceeds according to the addition–elimination mechanism (A–E) and allowed us to rationalize the stereochemical course and thermodynamic control of this reaction. Taking into account the Westheimer’s rules (apical attack, apical departure, pseudorotation, microscopic reversibility), we formulated the most likely mechanism of the alkaline hydrolysis of fluorophosphorinans, which is presented in Scheme 22. Thus, the attack of a hydroxyl anion on phosphorus in *cis*-**6**(**F**) results in formation of the first trigonal bipyramidal intermediate TBPI-A with apical fluorine and OH group and diequatorial six-membered ring. It decomposes slowly with inversion of configuration to thioacid *trans*-**9**, which is the minor product of this reaction. The fast Berry pseudorotation of TBPI-A leads to TBPI-B, which, in turn, undergoes permutational isomerization to TBPI-C and TBPI-D. Decomposition of the latter by departure of HF is virtually barrierless (see Supplementary Materials, Figure S7) and leads to the thermodynamically more stable thioacid *cis*-**9** with retention of configuration. This thioacid is the major and stable product. In the case of hydrolysis of *trans*-**6**(**F**), the more stable thioacid *cis*-**9** is formed from the first TBPI-A’ with inversion of configuration. The thioacid *trans*-**9** as the minor product of hydrolysis is formed after three consecutive pseudorotations with retention of P-configuration. As the phosphorane intermediate TBPI-B (and TBPI-B’) with the apical S are energetically unstable and could not be localized, it is possible that the transformation of TBPI-A into TBPI-C can take place directly via the tetragonal pyramidal intermediate (which may be a 2nd order saddle point) transiently formed from TBPI-A in the process of pseudorotation.

## 3. Materials and Methods

### 3.1. General Experimental Details

Commercial grade reagents and solvents were used without further purification except as indicated below. THF and diethyl ether were distilled over Na/benzophenone prior to use. Column chromatography was performed using silica gel (70–230 mesh). The routine ^1^H-NMR spectra were recorded at 60 and 80 MHz unless stated otherwise. Chemical shifts are quoted in parts per million (ppm) and reported relative to TMS as an internal standard. ^31^P-NMR spectra were obtained with a spectrometer operating at 24.3 MHz using 85% phosphoric acid as external standard. A heteronuclear spin decoupler was used for precise ^31^P chemical shift determinations. Diastereomeric purities were determined from integrated ^1^H- and ^31^P-NMR spectra and GLPC analysis. Boiling and melting points are uncorrected.

### 3.2. Synthetic Procedures

#### 3.2.1. *Trans*-2-Chloro-4-methyl-1,3,2-dioxaphosphorinan-2-thione **6**(**Cl**)

Procedure A. To a solution of cyclic *trans*-chlorophosphite **7** (6.95 g, 45 mmol) in ether (30 mL) was added acetylsulfonyl chloride (5 g, 45 mmol) at 0 °C. The reaction mixture was stirred at room temperature for 1 h. The solvent was evaporated and the residue was distilled under reduced pressure to give 7.7 g (91.4% yield) of the diastereomerically pure *trans*-**6**(**Cl**): bp. 78–80 °C; $n_{D}^{20}$ 1.5220; ^1^H-NMR (CCl_4_, 270 MHz) δ 1.26 (dd, 3H, ^3^*J*_CH3-H_ = 6.2 Hz, ^4^*J*_CH3-P_ = 2.6 Hz, CH_3_); ^31^P-NMR (C_6_H_6_) δ 59.0.Anal. Calcd. for C_4_H_8_O_2_PSCl: C, 25.74; H, 4.26; P, 16.59. Found: C, 25.80; H, 4.07; P, 16.55.

Procedure B. To a 1:1 mixture of cyclic *cis*- and *trans*-thiophosphites **8** (3.04 g, 20 mmol) in benzene (25 mL), sulfuryl chloride (2.7 g, 20 mmol) was added dropwise at 0 °C. The reaction mixture was stirred for 1 h at room temperature. After removal of solvent, the crude product was distilled under reduced pressure to afford 2.9 g (80% yield) of *trans*-**6**(**Cl**) (100% dp, δ_P_ 59.0): bp. 67 °C (0.01 mmHg); $n_{D}^{20}$ 1.5220. Anal. Calcd. for C_4_H_8_O_2_PSCl: C, 25.74; H, 4.26; P, 16.59. Found: C, 25.75; H, 4.52; P, 16.68.

#### 3.2.2. *Trans*-2-Bromo-4-methyl-1,3,2-dioxaphosphorinan-2-thione **6**(**Br**)

To a solution of cyclic thiophosphites **8** (73% *cis*-**8** and 27% *trans*-**8**) in benzene (20 mL), bromine (3.2 g, 20 mmol) was added dropwise at 30 °C. The reaction mixture was left overnight at room temperature. The reaction solution was washed with a few mL of sodium sulfite, then water, and then it was dried. After removal of benzene, the crude reaction product as a single isomer (^31^P-NMR assay) was distilled giving 3.9 g (85% yield) of pure *trans*-bromide **6**(**Br**), which after solidification was recrystallized from petroleum ether: bp. 82 °C (0.05 mmHg); $n_{D}^{20}$ 1.5562; mp. 41–43 °C; ^1^H-NMR (CCl_4_, 60 MHz) δ 1.47 (dd, 3H, ^3^*J*_CH3-H_ = 6.9 Hz, ^4^*J*_CH3-P_ = 2.9 Hz, 4-CH_3_); ^31^P-NMR (C_6_H_6_) δ 43.8. Anal. Calcd. for C_4_H_8_O_2_PSBr: C, 20.79; H, 3.49; P, 13.41. Found: C, 20.81; H, 3.43; P, 13.14.

#### 3.2.3. Reaction of Cyclic Phosphorothioic Acid *trans*-**9** with Phosphorus Pentachloride

To phosphorus pentachloride (1.04 g, 5 mmol) suspended in benzene (25 mL), thioacid *trans*-**9** (0.84 g, 5 mmol) was added dropwise at 0–5 °C. The reaction mixture was stirred for 1 h at this temperature and for the next 1 h at room temperature. After removal of volatile reaction products, the residue was analysed by ^31^P-NMR spectra. Based on the chemical shift values, intensity and shape of the P resonance signals, three compounds were identified as being the reaction products:

(1) thiophosphoryl chloride *trans*-**6**(**Cl**), 60%, δ_P_ 59.0 ppm;
(2) thiophosphoryl chloride *trans*-**5**(**Cl**), 14%, δ_P_ 3.5 ppm;
(3) unsymmetrical dithiopyrophosphate *trans*-*cis*-**10**, 21%, δ_P_ 45.7 and 50.0 ppm.

Distillation of the above mixture under reduced pressure gave 0.37g (40% yield) of *trans*-**6**(**Cl**), bp.78 °C (0.05 mmHg), $n_{D}^{20}$ 1.5218.

#### 3.2.4. Reaction of Cyclic Phosphorothioic Acid *cis*-**9** with Phosphorus Pentachloride

According to the procedure described above thioacid *cis*-**9** (0.84 g, 5 mmol) and PCl_5_ (1.04 g, 5 mmol) gave the crude reaction product, which according to ^31^P-NMR spectra consisted of three compounds:

1) thiophosphoryl chloride *cis*-**6**(**Cl**), 60%, δ_P_ 58.0 ppm;
2) thiophosphoryl chloride *trans*-**6**(**Cl**), 21%, δ_P_ 59.0 ppm;
3) unsymmetrical dithiopyrophosphate *trans*-*cis*-**10**, 19%, δ_P_ 45.7 and 50.0 ppm.

Upon fractional distillation a fraction boiling at 74 °C (0.05 mmHg) was collected, which contained *cis*-**6**(**Cl**) (95%) and *trans*-**6**(**Cl**) (5%); 0.30 g (32% yield).

#### 3.2.5. *Trans*-2-Fluoro-4-methyl-1,3,2-dioxaphosphorinan **11**

To chlorophosphite **7** (7.72 g, 50 mmol), antimony trifluoride (8.94 g, 50 mmol) was added portion wise at a temperature around 0–5 °C. The reaction mixture was then stirred at 45 °C for 0.5 h and distilled to give 6g (88% yield) of *trans*-fluorophosphite **11** containing 5% of *cis*-**11**; bp. 38 °C (11 mmHg); $n_{D}^{20}$ 1.4165. ^1^H-NMR (CCl_4_, 80 MHz) δ 1.26 (d, 3H, ^3^*J*_CH3-H_ = 6.2 Hz, CH_3_), 1.9 (m, 2H, CH_2_ at C_5_); 4.2 (m, 3H, H at C_4_, CH_2_ at C_6_); ^31^P-NMR (C_6_H_6_) δ 118.8 (^1^*J*_P-F_ = 1156 Hz, *trans*-**11**); 120.0 (^1^*J*_P-F_ = 1178 Hz, *cis*-**11**) Anal. Calcd. for C_4_H_8_PF: C, 34.89; H, 5.84; P, 22.43. Found: C, 34.88; H, 5.78; P, 22.85.

#### 3.2.6. *Cis-*2-Fluoro-4-methyl-1,3,2-dioxaphosphorinan-2-thione **6**(**F**)

To a solution of *trans*-fluorophosphite **11** (4.14 g, 30 mmol) in ether (20 mL), acetylsulfenyl chloride (3.32 g, 30 mmol) was added dropwise at a temperature around 0 °C and for additional 1 h at room temperature. After removal of solvent, the crude reaction product was distilled under reduced pressure to give 5.1 g (80% yield) of *cis*-**6**(**F**) (895% dp), which after solidification was recrystallized from petroleum ether: bp. 61 °C (0.1 mmHg); $n_{D}^{20}$ 1.4770; mp. 33–34 °C (*cis*-**6**(**F**)); ^1^H-NMR (CDCl_3_, 80 MHz) δ 1.46 (dd, 3H, ^3^*J*_CH3-H_ = 6.2 Hz, ^4^*J*_CH3-P_ = 2.6 Hz, CH_3_); ^31^P-NMR (CH_3_CN, 24.3 MHz) δ 54.0 (^1^*J*_P-F_ = 1086 Hz). Anal. Calcd. for C_4_H_8_O_2_PSF: C, 28.23; H, 4.74; P, 18.20. Found C, 28.17; H, 4.85; P, 17.97.

#### 3.2.7. *Trans*-2-Fluoro-4-methyl-1,3,2-dioxaphosphorinan-2-thione **6**(**F**)

Thiophosphoryl bromide *trans*-**6**(**Br**) (1.14 g, 5 mmol) was dissolved in acetonitrile (10 mL) and stirred with ammonium fluoride (1.48 g, 5 mmol) at 40 °C. Progress of the bromide–fluoride exchange was controlled by ^31^P-N MR spectra. After 24 h and standard isolation procedure, the crude reaction product was distilled under reduced pressure to give 0.40 g (44% yield) of *trans*-**6**(**F**) with 94% diastereomeric purity: bp. 80 °C (0.005 mmHg); $n_{D}^{20}$ 1.4874. *Trans*-**6**(**F**) with 100% dp was obtained by preparative gas chromatography. ^1^H-NMR (CDCl_3_, 80 MHz) δ 1.48 (dt, 3H, ^3^*J*_CH3-H_ = 6.5 Hz, ^4^*J*_P-CH3_ = 0.9 Hz, ^4^*J*_CH3-H_ = 0.9 Hz); ^31^P-NMR (CH_3_CN, 24.3 MHz) δ 55.0 (^1^*J*_P-F_ = 1094 Hz).

#### 3.2.8. General Procedure for Alkaline Hydrolysis of 2-Halogeno-4-methyl-1,3,2-dioxaphosphorinan-2-thiones **6**(**Cl**,**Br**,**F**)

To sodium hydroxide (0.4 g, 10 mmol) dissolved in water (20 mL) and dioxane (13 mL), cyclic thiophosphoryl halogenide **6** (5 mmol, 0.85, 0.93, 1.16 g for Cl, Br and F, respectively) in dioxane (5 mL) was added dropwise at 0 °C. The reaction mixture was stirred for 2 h at room temperature. After removal of water and dioxane, hot acetone was added to the residue. Then, the organic phase was filtered, concentrated and analyzed by ^31^P-NMR spectra. The sodium salts of diastereomeric thioacids **9** were converted via free thioacids to the corresponding dicyclohexyl ammonium salt. Their ratio was determined from proton decoupled ^31^P-NMR spectra. The following chemical shifts were recorded for the salts of thioacids **9**: *cis*-**9**Na, δ_P_ = 54.0 ppm; *trans*-**9**Na, δ_P_ = 50.5 ppm; *trans*-**9DCHAH**, δ_P_ = 50.5 ppm; *cis*-**9DCHAH**, δ_P_ = 53.5 ppm, and were in agreement with the literature data [15,19]. Hydrolysis of *trans*-**6**(**Cl**) is exemplifying the above general procedure.

#### 3.2.9. Alkaline Hydrolysis of 2-Chloro-4-methyl-1,3,2-dioxaphosphorinan-2-thione **6**(**Cl**)

Thiophosphoryl chloride *trans*-**6**(**Cl**) (0.94 g, 5 mmol) in dioxane (5 mL) was added dropwise to a solution of sodium hydroxide (0.4 g, 10 mmol) in water (25 mL) and dioxane (15 mL) at a temperature of about 2 °C. The reaction mixture was stirred for 2 h at room temperature. After evaporation of solvents in vacuo, the solid residue was dissolved in hot acetone. Then, the filtered solution was evaporated to give the sodium salts of *trans*-**9Na** and *cis*-**9Na** in a ratio of 92:8. The sodium salts obtained were dissolved in water (5 mL) and added to a solution of 25% hydrochloric acid. The water phase was extracted with CHCl_3_ (4 × 5 mL), dried over MgSO_4_ and evaporated to give the crude thioacid**9**. It was dissolved in ether and dicyclohexylamine was added. After removal of ether, the crude dicyclohexylammonium salt, *trans*-**9DCHAH**, was obtained (1.35 g, 80% yield) with 92% diastereomeric purity, mp. 206–210 °C; ^31^P-NMR (C_6_H_6_, 24.3 MHz) δ 50.5 (δ_P_ 53.5 for *cis*-salt). Anal. Calcd. for C_16_H_32_O_3_PSN: C, 54.99; H, 9.23; P, 8.86. Found: C, 55.07; H, 9.38; P, 8.92.

#### 3.2.10. General Procedure for Methanolysis of 2-Halogeno-4-methyl-1,3,2-dioxaphosphorinan-2-thiones **6**(**Cl**,**Br**,**F**)

To a solution of sodium methoxide (0.069 g, 3 mmol Na, in methanol, 7 mL) in benzene (20 mL) (or CH_3_CN and DMF), cyclic thiophosphoryl halogenide **6** (3 mmol, 0.51, 0.56, 0.69 g for Cl, Br and F, respectively) was added at ca. 0 °C. The reaction mixture was stirred for an additional 2 h. Sodium halogenide that precipitated was removed by washing the reaction solution, and it was then dried and evaporated. The residue was analyzed by ^31^P-NMR to determine the ratio of the diastereomeric methyl esters **12** formed (*trans*-**12**, δ_P_ = 63.5 ppm, *cis*-**12**, δ_P_ = 61.5 ppm). The pure esters **12** were obtained by distillation under reduced pressure. Methanolysis of *trans*-**6**(**Cl**) exemplifies the above general procedure.

#### 3.2.11. Methanolysis of 2-Chloro-4-methyl-1,3,2-dioxaphosphorinan-2-thione **6**(**Cl**)

To a solution of sodium methoxide (0.069 g, 3 mmol in methanol, 1.7 mL), cyclic thiophosphoryl chloride *trans*-**6**(**Cl**) (0.56 g, 3 mmol) in benzene (75 mL) was added dropwise at around 0–5 °C. The reaction mixture was stirred for 2 h. The reaction solution was washed with a few mL of water, then dried and evaporated. The ^31^P-NMR spectrum of the residue revealed the presence of *trans*-ester **12** and *cis*-ester **12** in a ratio of 93:7. Distillation of the crude product gave 0.38 g (70% yield) of analytically pure ester **12** (mixture of *trans*- and *cis*- isomers): bp. 78–80 °C (0.3 mmHg), $n_{D}^{20}$ 1.4923. Anal. Calcd. for C_5_H_11_O_3_PS: C, 32.96; H, 6.08; P, 17.00. Found: C, 32.47; H, 6.00; P, 17.00.

#### 3.2.12. General Procedure for Aminolysis of 2-Halogeno-4-methyl-1,3,2-dioxaphosphorinan-2-thiones **6**(**Cl**,**Br**,**F**)

To a solution of dimethylamine (1.13 g, 25 mmol) in ether (25 mL), cyclic thiophosphorylhalogenide **6** (10 mmol, 1.87, 2.31, 1.70 g for Cl, Br and F, respectively, in ether, 15 mL) was added dropwise at ca. 0 °C. The reaction mixture was stirred for 1 h. The precipitated dimethylammonium halogenide was filtered off. The filtrate was evaporated and the crude product was analyzed by gas chromatography. Aminolysis of *trans*-**6**(**Cl**) exemplifies the above general procedure.

#### 3.2.13. Aminolysis of 2-Chloro-4-methyl-1,3,2-dioxaphosphorinan-2-thione **6**(**Cl**)

To a solution of dimethylamine (1.1 g, 25 mmol) in ether (25 mL), cyclic thiophosphoryl chloride *trans*-**6**(**Cl**) was added dropwise at 0 °C. After stirring the reaction mixture for 1 h the precipitated ammonium chloride was filtered off and the filtrate evaporated. Distillation of the crude reaction product gave the pure amide *trans*-**13** (1.6 g, 76% yield): bp. 60°C (0.05 mmHg), $n_{D}^{20}$ 1.5031, mp. 35–36.5 °C, δ_P_ = 75.0 ppm. Anal. Calcd. for C_6_H_14_O_2_PSN: C, 36.91; H, 7.25; P, 15.87. Found: C, 37.15; H, 7.00; P, 16.04.

#### 3.2.14. Synthesis of 2-*N*,*N*-Dimethylamino-4-methyl-1,3,2-dioxaphosphorinan-2-thione **13**

To a solution of cyclic amidophosphite **14** (*cis*-**14**, 80% and *trans*-**14**, 20%) (5,5 g, 33 mmol) in ether (20 mL), elemental sulfur (1.1 g, 33 mmol) was added at 0 °C. After stirring for 1 h, ether was evaporated and the crude product was distilled under reduced pressure to afford 4.9 g (74% yield) of *trans*-**13** 82% dp (GC assay): bp. 65 °C (0.05 mmHg), $n_{D}^{20}$ 1.5030, δ_P_ = 75.0 ppm. Anal. Calcd. for C_6_H_14_NO_2_PS: C, 36.91; H, 7.23; P, 15.87. Found: C, 37.20; H, 7.40; P, 15.82.

#### 3.2.15. Theoretical Methods

All quantum mechanical calculations were performed using the Gaussian 16 suite of programs [39]. Geometries of the model compounds were optimized using two DFT methods: the hybrid B3LYP density functional [40] corrected for dispersion interactions using Grimme GD3 empirical term [41], with the Def2TZVP basis set [42] in aqueous solution. SCRF calculations in water were performed using CPCM model with UFF atomic radii as implemented in Gaussian 16 [39]. All stationary points were identified as stable minima by frequency calculations. The vibrational analysis provided thermal enthalpy and entropy corrections at 298 K within the rigid rotor/harmonic oscillator/ideal gas approximation [39]. Thermochemical corrections were scaled by a factor of 0.99.

## Figures and Tables

**Table 1:** ^1^H-NMR data for trans-2-chloro-4-methyl-1,3,2-dioxaphosphorinan-2-thione **6**(**Cl)** in carbon tetrachloride solution at 270 MHz; atom numbering in the ring shown below.

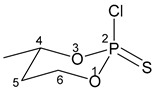								
Proton	Chemical Shift (δ, ppm)	Coupling Constants (Hz) to:						
		CH_3_	5eq	5ax	6eq	6ax	4ax	P
CH_3_	1.26	n/a	-	-	-	-	6.2	2.6
5eq	1.59	-	n/a	14.8	1.85	2.6	2.6	1.25
5ax	1.85	-	14.8	n/a	4.8	11.5	11.5	-
6eq	4.17	-	1.85	4.8	n/a	11.5	-	29.3
6ax	4.30	-	2.6	11.5	11.5	n/a	-	4.4
4ax	4.54	6.2	2.6	11.5	-	-	n/a	3.3

**Table 2:** Effect of reaction medium on percentage of *cis*- and *trans*-isomers of thioacid **9** obtained in hydrolysis of thiophosphoryl fluoride *cis*-**6**(**F**).

No	Reaction Medium	Sodium Salt	
		*cis*-9(Na) (%) Retention	*trans*-9(Na) (%) Inversion
1	NaOH, water-dioxane (1:1)	66	34
2	NaOH, water-DMF (1:1)	66	34
3	NaOH, water-DMF (1:1), Et_4_NBr	54	36
4	NaOH, water-dioxane (1:1), NH_4_F	70	30
5	KOH, water-dioxane (1:1)	64	36

**Table 3:** Effect of diastereomeric purity of thiophosphoryl fluoride *trans*-**6**(**F**) on percentage of *cis*- and *trans*-isomers of thioacid **9** sodium salt.

No	Diastereomeric Purity of *trans*-6(F), dp (%)	Sodium Salt	
		*cis*-9(Na) (%) Inversion	*trans*-9(Na) (%) Retention
1	71	78	22
2	89	77	25
3	90	74	26
4	94	73	27

**Table 4:** Effects of solvents on percentage of *cis*- and *trans*-thiophosphates **12** obtained in methanolysis of thiophosphoryl fluoride *cis*-**6**(**F**).

No.	Solvent	Thionophosphate 12	
		*cis*-12	*trans*-12
1	C_6_H_6_	84	16
2	DMF	87	13
3	MeCN	88.5	11.5
4	MeOH	88	12

**Table 5:** Free energies relative to the sum of the free energies of free substrates for the reaction of *cis*-**6**(**F**) with OH^−^.

	a		b		c	
	ΔH	ΔG	ΔH	ΔG	ΔH	ΔG
complex of substrates	−5.3	2.0	−6.7	1.1	−6.7	1.1
transition state 1	1.2	10.7	2.8	12.4	8.6	18.8
intermediate	−23.2	−12.4	−19.8	−9.9	−28.2	−17.7
transition state 2	−18.4	−7.9	−14.4	−2.8	−27.4	−16.5
complex of products	−25.6	−17.5	−58.1	−49.5	−58.1	−49.5
free products	−19.9	−18.6	−42.0	−41.8	−42.0	−41.8

**Table 6:** Free energies relative to the sum of the free energies of free substrates for the reaction of *trans*-**6**(**F**) with OH^−^.

	a		b		c	
	ΔH	ΔG	ΔH	ΔG	ΔH	ΔG
complex of substrates	−4.3	2.4	−5.0	2.5	−5.0	2.5
transition state 1	−2.5	7.4	4.7	14.5	4.4	13.7
intermediate	−21.4	−11.8	−18.4	−8.3	−29.4	−18.7
transition state 2	−14.3	−4.2	−11.2	0.3	−27.9	−16.9
complex of products	−25.3	−17.3	−30.6	−19.4	−58.7	−50.3
free products	−23.0	−20.5	−45.8	−44.2	−45.8	−44.2

## Data Availability

The data presented in this study are available on request from the corresponding author.

## Supplementary Materials

The following are available online, Figure S1: Geometries of *trans* and *cis* isomers of 2-chloro-4-methyl-1,3,2-dioxaphosphorinan-2-thione 6(Cl); green—chlorine, orange—phosphorus, yellow—sulfur, red—oxygen, grey—carbon, white—hydrogen, Table S1: Relative Gibbs free energies (kcal/mol) of the most stable conformers of *cis*- and *trans*-2-chloro-4-methyl-1,3,2-dioxaphosphorinan-2-thione 6(Cl) (for structures see Figure S1), Figure S2: Geometries of cis and trans conformers of 2-fluoro-4-methyl-1,3,2-dioxaphosphorinan-2-thione 6(F); cyan—fluorine, remaining colours as in Figure S1, Table S2: Relative Gibbs free energies (kcal/mol) of all isomers of 2-fluoro-4-methyl-1,3,2-dioxaphosphorinan-2-thione 6(F) (for structures see Figure S2), Figure S3: Structures of *cis* and *trans* conformers of 2-hydroxy-4-methyl-1,3,2-dioxaphosphorinan-2-thione 9, Table S3: Relative Gibbs free energies (kcal/mol) of all isomers of 2-hydroxy-4-methyl-1,3,2-dioxaphosphorinan-2-thione 9 (for structures see Figure S3). Figure S4: Crystal (a) and DFT (b) structures of *cis*-2-fluoro-4-methyl-1,3,2-dioxaphosphorinan-2-thione 6(F), Table S4: Comparison of X-ray [24] and DFT selected structural parameters for *cis*-2-fluoro-4-methyl-1,3,2-dioxaphosphorinan-2-thione 6(F) (bond distances in Å, angles in deg), Figure S5 and S6: Stationary points for the backside attack of hydroxyl anion on chlorothiophosphorinan 6(Cl), Table S5: Free energies relative to the sum of the free energies of free substrates for the reaction of *trans*- and *cis*-2-chloro-4-methyl-1,3,2-dioxaphosphorinan-2-thione 6(Cl) with OH^−^, Figure S7: Total free energy profiles(kcal/mol) for the three reaction pathways (a, b, c—see main text) of fluoride substitution in *cis*-6(F) (path b appears in two variants which differ only slightly, so the plots overlap each other).

## Sample Availability

Samples of compounds are not available from the authors.

## References

[1] Kolodiazhnyi O.I., Kolodiazhna A. (2017). Nucleophilic substitution at phosphorus: Stereochemistry and mechanisms. Tetrahedron Asymmetry.

[2] Michalski J., Mikołajczyk M., Omelańczuk J. (1965). Stereochemistry of nucleophilic displacement reaction at thiophosphoryl centre. An example of a Walden cycle involving phosphorus. Tetrahedron Lett..

[3] Michalski J., Mikołajczyk M., Młotkowska B., Omelańczuk J. (1969). Stereochemistry of nucleophilic substitution reaction at a thiophosphorylcentre—IV: Chemical proof of Walden inversion. Tetrahedron.

[4] Michalski J., Mikołajczyk M., Halpern A., Prószyńska K. (1966). Stereochemistry of nucleophilic displacement reactions at the thiophosphorylcentre. Chloride-chloride exchange at the asymmetric phosphorus atom in O-ethyl ethylphosphonochloridothionate. Tetrahedron Lett..

[5] Mikołajczyk M. (1967). Stereochemistry of nucleophilic displacement reactions at the thiophosphorylcentre—II: Hydrolysis of optically active o-ethyl ethylphosphonochloridothionate. Tetrahedron.

[6] Westheimer F.H. (1968). Pseudo-rotation in the hydrolysis of phosphate esters. Acc. Chem. Res..

[7] Bujnicki B., Drabowicz J., Mikołajczyk M. (2015). Acid catalyzed alcoholysis of sulfinamides: Unusual stereochemistry, kinetics and a question of mechanism involving sulfurane intermediates and their pseudorotation. Molecules.

[8] Mikołajczyk M., Witczak M. (1977). Stereochemistry of organophosphorus cyclic compounds. Part 5. Synthesis and geometry of some 4,5-disubstituted 1,3,2-dioxaphospholan-2-thiones and stereochemistry of nucleophilic displacement reactions at phosphorus. J. Chem. Soc. Perkin Trans. 1.

[9] Denney D., Chang B.C., Conrad W.E., Denney D.Z., Edelman R., Powell R.L., White D.W. (1971). Exchange route to oxyphosphoranes. J. Am. Chem. Soc..

[10] Maryanoff B.E., Hutchins R.O., Maryanoff C.A., Allinger N.L., Eliel E.I. (1979). Stereochemical aspects of phosphorus-containing cyclohexanes. Topic in Stereochemistry.

[11] Wadsworth W.S. (1973). Effect of added salts on the stereochemistry of nucleophilic displacements at phosphorus in phosphate esters and their analogs. J. Org. Chem..

[12] Stec W., Mikołajczyk M. (1973). Stereochemistry of organophosphorus cyclic compounds—II: Stereospecific synthesis of cis- and trans 2-halogeno-2-oxo-4-methyl-1,3,2-dioxaphosphorinans and their chemical transformations. Tetrahedron.

[13] Mikołajczyk M., Krzywański J., Ziemnicka B. (1974). Synthesis of the Diastereomerically Pure trans-2-Halogeno-4-methyl-1,3,2,-dioxaphosphorinan-2-thiones and Conformational Studies. Phosphorus.

[14] Mikołajczyk M., Krzywański J., Ziemnicka B. (1975). Diastereomeric 2-fluoro-4-methyl-1,3,2-dioxaphosphorinan-2-thiones. Retention stereochemistry in nucleophilic substitution at thiophosphoryl phosphorus. Tetrahedron Lett..

[15] Mikołajczyk M., Gajl M., Blaszczyk J., Cypryk M., Gostynski B. (2020). Nucleophilic Substitution at Tetracoordinate Sulfur. Kinetics and Mechanism of the Chloride-Chloride Exchange Reaction in Arenesulfonyl Chlorides: Counterintuitive Acceleration of Substitution at Sulfonyl Sulfur by ortho-Alkyl Groups and Its Origin. Molecules.

[16] Aksnes G., Eriksen R., Mellingen K., Åkeson Å., Theorell H., Blinc R., Paušak S., Ehrenberg L., Dumanović J. (1967). Studies of Geometric Isomers of Cyclic Phosphites. Acta Chem. Scand..

[17] Bodkin C.L., Simpson P. (1971). The stereochemistry of 2-alkoxy-4-methyl-1,3,2-dioxaphosphorinans. J. Chem. Soc. B.

[18] Bodkin C.L., Simpson P. (1973). A stereochemical study of some reactions of compounds in the 4-methyl-1,3,2-dioxaphosph(V)orinan series. J. Chem. Soc. Perkin Trans. 2.

[19] Mikołajczyk M., Krzywanski J., Ziemnicka B. (1977). Stereochemistry of organophosphorus cyclic compounds. 6. Stereochemistry of the reaction between sulfenyl chlorides and trivalent phosphorus compounds. J. Org. Chem..

[20] Nifantev E.E., Nasonovski J.I. (1972). Stereochemical Aspect of 1,3-Butylenephosphite Reactions. Dokl. Akad. Nauk SSSR.

[21] Zwierzak A. (1967). Cyclic organophosphorus compounds. I. Synthesis and infrared spectral studies of cyclic hydrogen phosphites and thiophosphites. Can. J. Chem..

[22] Mikołajczyk M., Michalski J. (1964). Optically active O-ethyl ethylphosphonochloridothionates—A new route to optically active organophosphorus compounds. Chem. Ind..

[23] Mikołajczyk M., Łuczak J. (1972). Stereochemistry of organophosphorus cyclic compounds—I: Stereospecific synthesis of cis- and trans-2-hydroxy-2-thio(seleno)-4-methyl-1.3.2-Dioxaphosphorinans. Tetrahedron.

[24] Mikołajczyk M., Ziemnicka B., Karolak-Wojciechowska J., Wieczorek M. (1983). Stereochemistry of cyclic organophosphorus compounds. Part 13. Stereoisomerism in bis-(4-methyl-2-thioxo-1,3,2-dioxaphosphorinan-2-yl) oxide. Synthesis of diastereoisomers and their solution and solid state conformations. J. Chem. Soc. Perkin Trans. 2.

[25] Omelańczuk J., Kielbasiński P., Michalski J., Mikołajczak J., Mikołajczyk M., Skowrońska A. (1975). Reaction of oxophosphoranesulphenyl chlorides with phosphorus trichloride: A new synthesis of dialkylphosphorochloridothionates and their optically active analogues. Tetrahedron.

[26] Green M., Hudson R.F. (1959). Proceedings of the Chemical Society. August 1959. Proc. Chem. Soc..

[27] Schmutzler R. (1963). Chemie der Phosphorfluoride, VII. Synthese und Koordinationschemie der Fluorophosphite. Chem. Ber..

[28] Miller A., Wieczorek M.W., Karolak-Wojciechowska J., Mikołajczyk M., Ziemnicka B. (1981). The structure of cis-2-fluoro-4-methyl-1,3,2-dioxaphosphorinane 2-sulphide. Acta Cryst. B.

[29] Nifantev E.E., Nasonovski J.I., Borisenko A.A. (1970). Stereoisomerism of Cyclic Acid Phosphites. Zh. Obsh. Khim..

[30] Harrison J.M., Inch T.D., Lewis G.J. (1974). Use of carbohydrate derivatives for studies of phosphorus stereochemistry. Part III. Stereochemical course of nucleophilic displacements of 2-substituents in 1,3,2-dioxaphosphorinan-2-ones and related compounds. J. Chem. Soc. Perkin Trans. 1.

[31] Potrzebowski M.J., Bujacz G.D., Bujacz A., Olejniczak S., Napora P., Helinski J., Ciesielski W., Gajda J. (2006). Study of molecular dynamics and the solid state phase transition mechanism for unsymmetrical thiopyrophosphate using X-ray diffraction, DFT calculations and NMR spectroscopy. J. Phys. Chem. B.

[32] Masnabadi N. (2020). DFT Study and NBO Analysis of Conformation Properties of 2,5,5-Trimethyl-1,3,2-Dioxaphosphinane 2-Selenide and Their Dithia and Diselena Analogous. J. Sci..

[33] Kanzari-Mnallah D., Efrit M.L., Pavlíček J., Vellieux F., Boughzala H., Akacha A.B. (2019). Synthesis, Conformational Analysis and Crystal Structure of New Thioxo, Oxo, Seleno Diastereomeric Cyclophosphamides Containing 1,3,2-dioxaphosphorinane. Curr. Org. Chem..

[34] Vafaei-Nezhada M., Ghiasib R., Shafiei F. (2020). Conformational Analysis of 2-halo-1,3,2-dioxaphosphinanes: A Density Functional Theory (DFT) Investigation. Chem. Methodol..

[35] van Bochove M.A., Swart M., Bickelhaupt F.M. (2006). Nucleophilic Substitution at Phosphorus (SN2@P): Disappearance and Reappearance of Reaction Barriers. J. Am. Chem. Soc..

[36] van Bochove M.A., Swart M., Bickelhaupt F.M. (2009). Stepwise Walden inversion in nucleophilic substitution at phosphorus. Phys. Chem. Chem. Phys..

[37] Bento A.P., Bickelhaupt F.M. (2008). Nucleophilicity and Leaving-Group Ability in Frontside and Backside SN2 Reactions. J. Org. Chem..

[38] Mislow K. (1970). Role of pseudorotation in the stereochemistry of nucleophilic displacement reactions. Acc. Chem. Res..

[39] Frisch M.J., Trucks G.W., Schlegel H.B., Scuseria G.E., Robb M.A., Cheeseman J.R., Scalmani G., Barone V., Petersson G.A., Nakatsuji H. (2016). Gaussian 16, Revision C.01.

[40] Becke A.D. (1993). Density-functional thermochemistry. III. The role of exact exchange. J. Chem. Phys..

[41] Grimme S., Jens A., Ehrlich S., Krieg H. (2010). A consistent and accurate ab initio parametrization of density functional dispersion correction (DFT-D) for the 94 elements H-Pu. J. Chem. Phys..

[42] Weigend F., Ahlrichs R. (2005). Balanced basis sets of split valence, triple zeta valence and quadruple zeta valence quality for H to Rn: Design and assessment of accuracy. Phys. Chem. Chem. Phys..

