# Supplementary material for: Nucleophilic Substitution at Tetracoordinate Phosphorus. Stereochemical Course and Mechanisms of Nucleophilic Displacement Reactions at Phosphorus in Diastereomeric cis- and trans-2-Halogeno-4-methyl-1,3,2-dioxaphosphorinan-2-thiones: Experimental and DFT Studies
Source: Molecules. 2021 Jun 15;26(12):3655. doi: 10.3390/molecules26123655 (PMC8232685; doi:10.3390/molecules26123655)
Supplement: Supplementary file 1 [file molecules-26-03655-s001.zip › molecules-1197268-supplementary.pdf]

Nucleophilic Substitution at Tetracoordinate Phosphorus. Stereochemical Course and Mechanisms of Nucleophilic Displacement Reactions at Phosphorus in Diastereomeric *cis*- and *trans*-2-Halogeno-4-methyl-1,3,2-dioxaphosphorinan-2-thiones: Experimental and DFT Studies

Marian Mikołajczyk[1,*], Barbara Ziemnicka[1], Jan Krzywański[1], Marek Cypryk[2,*], Bartłomiej Gostyński[2]

Supplementary Materials

Conformational analysis

trans-**6(Cl)**

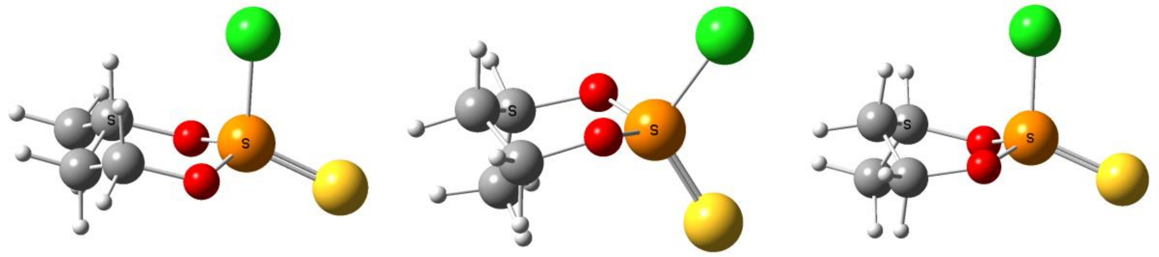

(chair) (Cl-ax,Me-eq) (chair) (Cl-eq,Me-ax) (twisted boat) (Cl-ax,Me-ax)

cis-**6(Cl)**

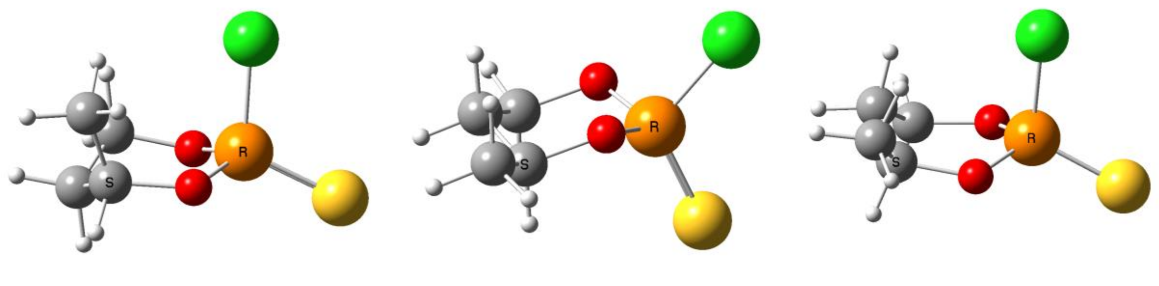

(twisted chair) (Cl-ax,Me-ax) (chair) (Cl-eq,Me-eq) (boat) (Cl-ax,Me-eq)

Figure S1. Geometries of *trans* and *cis* isomers of 2-chloro-4-methyl-1,3,2-dioxaphosphorinan-2-thione **6(Cl)**; green – chlorine, orange – phosphorus, yellow – sulfur, red – oxygen, grey – carbon, white - hydrogen.

Table S1. Relative Gibbs free energies (kcal/mol) of the most stable conformers of *cis*- and *trans*-2-chloro-4-methyl-1,3,2-dioxaphosphorinan-2-thione **6(Cl)** (for structures see Fig. S1).

relative geometry	ΔG_{rel}
trans (chair) (Cl-ax,Me-eq)	0
trans (chair) (Cl-eq,Me-ax)	5.6
trans (twisted boat) (Cl-ax,Me-ax)	3.4
cis (twisted chair) (Cl-ax,Me-ax)	3.9
cis (chair) (Cl-eq,Me-eq)	2.7
cis (boat) (Cl-ax,Me-eq)	2.5

trans-**6(F)**

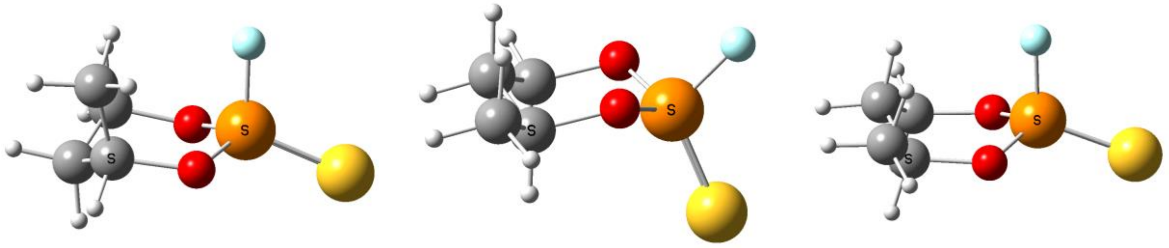

(chair) (F-ax,Me-ax) (chair) (F-eq,Me-eq) (boat) (F-ax,Me-eq)

cis-**6(F)**

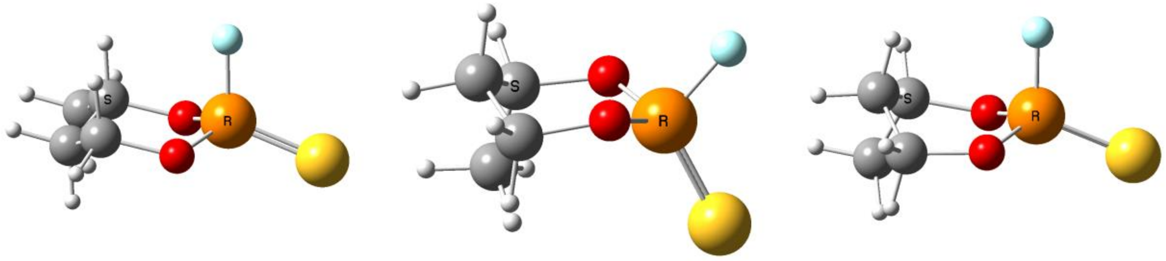

(chair) (F-ax,Me-eq) (chair) (F-eq,Me-ax) (boat) (F-ax,Me-ax)

Figure S2. Geometries of *cis* and *trans* conformers of 2-fluoro-4-methyl-1,3,2-dioxaphosphorinan-2-thione **6(F)**; cyan – fluorine, remaining colours as in Figure S1.

Table S2. Relative Gibbs free energies (kcal/mol) of all isomers of 2-fluoro-4-methyl-1,3,2-dioxaphosphorinan-2-thione **6(F)** (for structures see Fig. S2).

relative configuration	ΔG_{rel}
cis (chair) (F-ax,Me-eq)	0
cis (chair) (F-eq,Me-ax)	4.1
cis (boat) (F-ax,Me-ax)	2.3
trans (chair) (F-ax,Me-ax)	2.0
trans (chair) (F-eq,Me-eq)	2.5
trans (boat) (F-ax,Me-eq)	1.3

trans-**9**

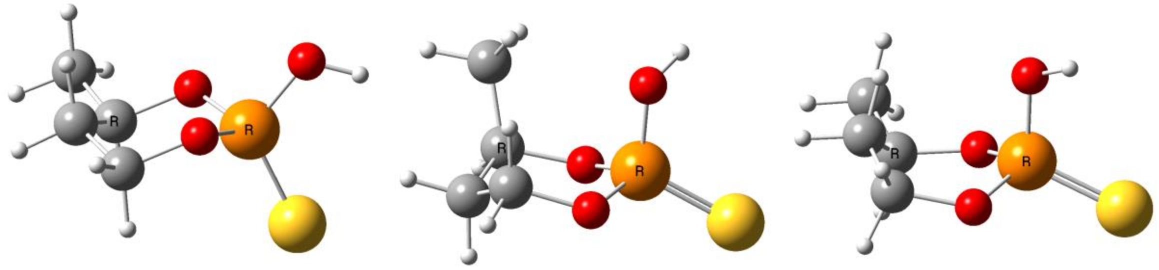

trans (chair) (OH-eq,Me-eq) *trans* (chair) (OH-ax,Me-ax) *trans* (boat) (OH-ax,Me-eq)

cis-**9**

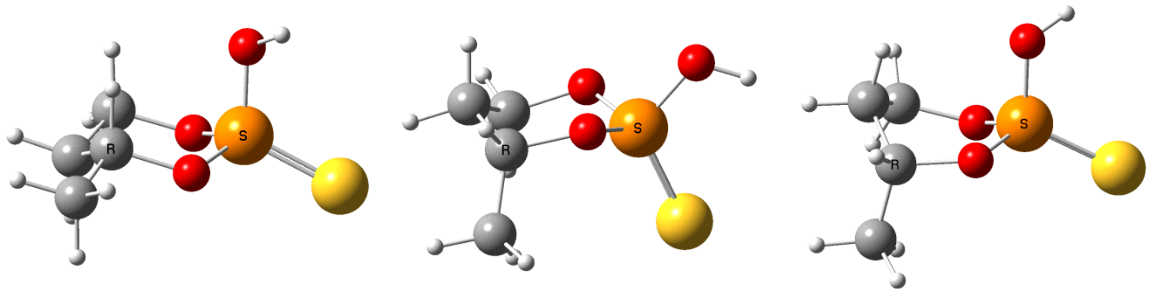

cis (chair) (OH-ax,Me-eq) *cis* (chair) (OH-eq,Me-ax) *cis* (boat) (OH-ax,Me-ax)

Figure S3. Structures of *cis* and *trans* conformers of 2-hydroxy-4-methyl-1,3,2-dioxaphosphorinan-2-thione **9**.

Table S3. Relative Gibbs free energies (kcal/mol) of all isomers of 2-hydroxy-4-methyl-1,3,2-dioxaphosphorinan-2-thione **9** (for structures see Fig. S3).

relative configuration	ΔG_{rel}
cis (chair) (OH-ax,Me-eq)	0
cis (chair) (OH-eq,Me-ax)	3.2
cis (boat) (OH-ax,Me-ax)	3.3
trans (chair) (OH-ax,Me-eq)	2.3
trans (chair) (OH-ax,Me-ax)	0.7
trans (boat) (OH-ax,Me-ax)	1.9

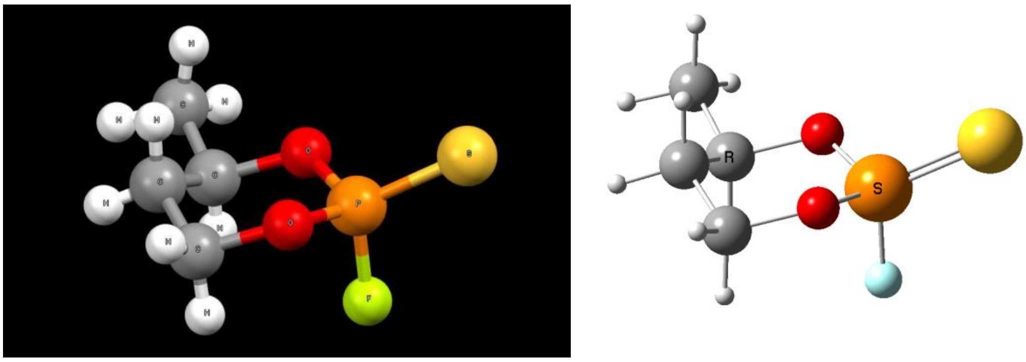

(a) (b)

Figure S4. Crystal (a) [ref. 28] and DFT (b) structures of *cis*-2-fluoro-4-methyl-1,3,2-dioxaphosphorinan-2-thione **6(F)**.

Table S4. Comparison of X-ray [ref. 28] and DFT selected structural parameters for *cis*-2-fluoro-4-methyl-1,3,2-dioxaphosphorinan-2-thione **6(F)** (bond distances in Å, angles in deg).

	X-ray [28]	DFT
Bonds		
P-S	1.887	1.910
P-F	1.558	1.583
P-O(Me)	1.552	1.576
P-O	1.555	1.579
Angles		
F-P-S	112.7	113.5
O(Me)-P-S	115.8	115.8
O(Me)-P-F	102.4	102.0
O-P-S	115.3	115.5
O-P-F	102.0	101.8
O-P-O(Me)	107.2	106.5

Study of substitution of halogen by hydroxide anion

Hydrolysis of 2-chloro-4-methyl-1,3,2-dioxaphosphorinan-2-thione **6(Cl)**

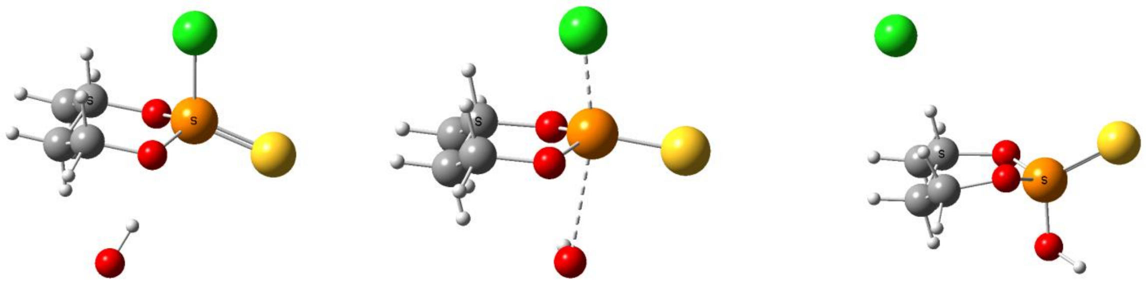

Complex of substrates Transition state Complex of products

Figure S5. Stationary points for the backside attack of hydroxyl anion on chlorothiophosphorinan *trans*-**6(Cl)**.

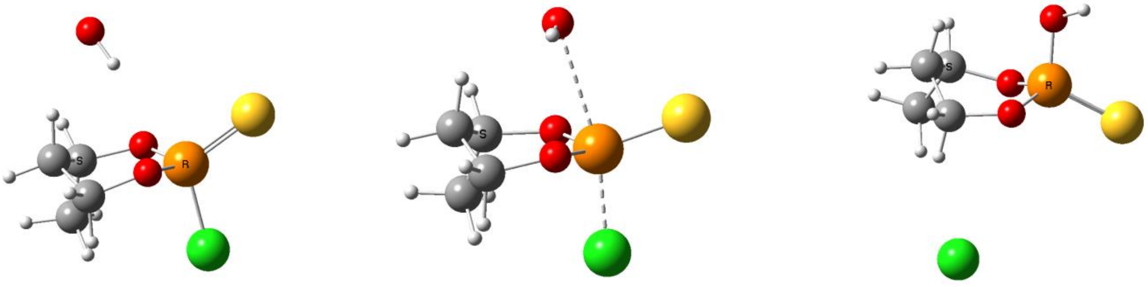

Complex of substrates Transition state Complex of products

Figure S6. Stationary points for the backside attack of hydroxyl anion on chlorothiophosphorinan *cis*-**6(Cl)**.

Table S5. Free energies relative to the sum of the free energies of free substrates for the reaction of *trans*- and *cis*-2-chloro-4-methyl-1,3,2-dioxaphosphorinan-2-thione **6(Cl)** with OH^-.

	***trans*-6(Cl)**		***cis*-6(Cl)**	
	ΔH	ΔG	ΔH	ΔG
complex of substrates	-5.4	2.2	-5.4	3.3
transition state	0.8	10.4	0.0	10.3
complex of products	-62.9	-56.4	-61.6	-54.8
free products	-62.7	-61.2	-59.0	-58.6

Hydrolysis of 2-fluoro-4-methyl-1,3,2-dioxaphosphorinan-2-thione **6(F)**

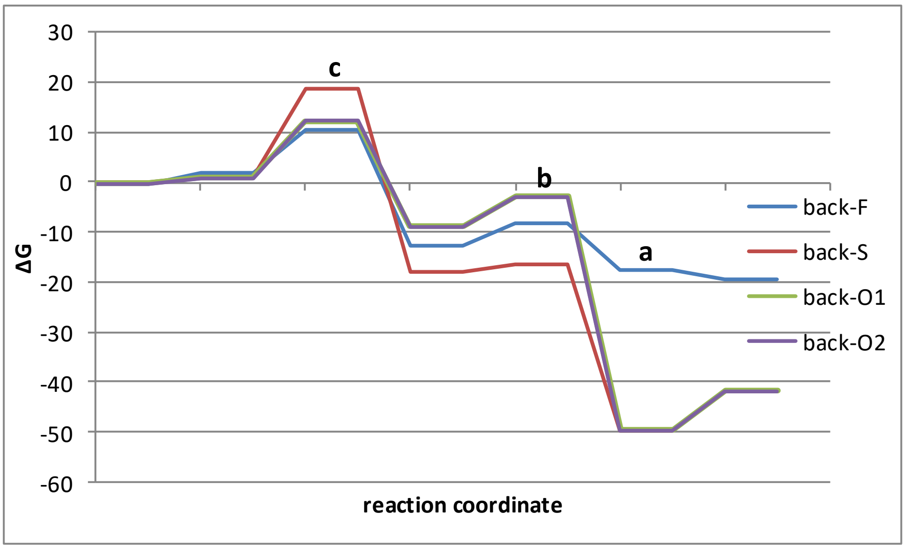

Figure S7. Total free energy profiles(kcal/mol) for the three reaction pathways (**a**, **b**, **c** – see Scheme 21 in the main text) of fluoride substitution in *cis*-**6(F)** (path **b** appears in two variants which differ only slightly, so the plots overlap each other).
